# Basophil Activation to Gluten and Non-Gluten Proteins in Wheat-Dependent Exercise-Induced Anaphylaxis

**DOI:** 10.3389/falgy.2022.822554

**Published:** 2022-02-25

**Authors:** Angelika Miriam Gabler, Julia Gebhard, Marie-Christin Norwig, Bernadette Eberlein, Tilo Biedermann, Knut Brockow, Katharina Anne Scherf

**Affiliations:** ^1^Leibniz-Institute for Food Systems Biology at the Technical University of Munich, Freising, Germany; ^2^Department of Dermatology and Allergy Biederstein, TUM School of Medicine, Technical University of Munich (TUM), Munich, Germany; ^3^Department of Bioactive and Functional Food Chemistry, Institute of Applied Biosciences, Karlsruhe Institute of Technology (KIT), Karlsruhe, Germany

**Keywords:** allergy, amylase/trypsin inhibitor, basophil activation test, gluten, proteomics, wheat

## Abstract

Wheat-dependent exercise-induced anaphylaxis (WDEIA) is a cofactor-induced wheat allergy. Gluten proteins, especially ω5-gliadins, are known as major allergens, but partially hydrolyzed wheat proteins (HWPs) also play a role. Our study investigated the link between the molecular composition of gluten or HWP and allergenicity. Saline extracts of gluten (G), gluten with reduced content of ω5-gliadins (G-ω5), slightly treated HWPs (sHWPs), and extensively treated HWPs (eHWPs) were prepared as allergen test solutions and their allergenicity assessed using the skin prick test and basophil activation test (BAT) on twelve patients with WDEIA and ten controls. Complementary sodium dodecyl-sulfate polyacrylamide gel electrophoresis (SDS-PAGE), high-performance liquid chromatography (HPLC), and mass spectrometry (MS) analyses revealed that non-gluten proteins, mainly α-amylase/trypsin inhibitors (ATIs), were predominant in the allergen test solutions of G, G-ω5, and sHWPs. Only eHWPs contained gliadins and glutenins as major fraction. All allergen test solutions induced significantly higher %CD63^+^ basophils/anti-FcεRI ratios in patients compared with controls. BAT using sHWPs yielded 100% sensitivity and 83% specificity at optimal cut-off and may be useful as another tool in WDEIA diagnosis. Our findings indicate that non-gluten proteins carrying yet unidentified allergenic epitopes appear to be relevant in WDEIA. Further research is needed to clarify the role of nutritional ATIs in WDEIA and identify specific mechanisms of immune activation.

## Introduction

Wheat-dependent exercise-induced anaphylaxis (WDEIA) is a cofactor-induced wheat allergy. It is generally considered to be rare. In Japanese adolescents, the prevalence of food-dependent exercise-induced anaphylaxis predominantly to wheat was 0.017%. However, as these patients tolerate wheat in the absence of association with cofactors, WDEIA may not be recognized in many patients and they are often given the diagnosis of idiopathic anaphylaxis instead (1–3).

Patients with WDEIA may react to intact gluten proteins and/or partially hydrolyzed wheat proteins (HWPs) (4–6). Besides others, Yokooji et al. and Hiragun et al. reported allergic reactions in patients with WDEIA to HWPs in facial soap (6, 7). HWPs are made of gluten subjected to chemical or enzymatic partial hydrolysis to obtain foaming and emulsifying properties for use in foods and cosmetics (6, 8, 9). Depending on the treatment, HWPs differ significantly from one another regarding their functional properties and molecular composition (8, 10–12). Partial hydrolysis may lead to exposure of pre-existent allergenic epitopes otherwise buried within protein aggregates or to the formation of new epitopes, e.g., through deamidation (6, 7). The increase in solubility of HWPs compared with native gluten also affects allergen passage through the skin or the small intestine (6, 7, 13).

About 80% of patients with WDEIA have specific IgE (sIgE) against ω5-gliadins, the major allergens in WDEIA (14), but sensitization to other wheat gluten proteins, such as high- and low-molecular-weight glutenin subunits (HMW-GS and LMW-GS) or α- and γ-gliadins has also been reported (15–20). Water- or salt-soluble non-gluten proteins, such as lipid-transfer proteins (LTPs) associated with baker's asthma, were also suggested to play a role in WDEIA (21–23). Pastorello et al. found sIgE against α-amylase/trypsin inhibitors (ATIs) in WDEIA patients' sera (24), but the role of nutritional non-gluten proteins as causative agents for WDEIA is currently underexplored.

Approaches to diagnose WDEIA include clinical history, skin prick test (SPT), measurement of sIgE against ω5-gliadins, and oral gluten challenge combined with cofactor as golden standard (4). Due to the risk of a serious anaphylactic reaction during the challenge tests, there is a need to establish alternatives. The *in vitro* basophil activation test (BAT) using well-defined allergen test solutions (ATSs) may be suitable, because basophil activation is directly related to the allergenicity of a test substance (25–28). The BAT is already used to diagnose and investigate IgE-mediated allergies, e.g., allergy against antibiotics (29) or bee and wasp venom (30). Schwager et al. evaluated the allergenic potential of natural and recombinant peanut oleosins using the BAT on peanut-allergic and peanut-sensitized patients in comparison with a control group. A complex cocktail of 12 antibodies was used to identify basophils. The activation marker of identified basophils was CD63 (31). The same group improved the BAT workflow for reliable results with a time saving approach to make it suitable for clinical routine. *Inter alia*, they compared the approach of Schwager et al. with a simplified approach using CD63 (activation marker) and CD203c and FcεRIα (identification markers). As they found no significant differences between the results of both strategies, they showed that the necessary simplification to make BAT applicable in clinical routine is possible and reliable. Furthermore, Behrends et al. used different peanut allergens in the BAT, such as oleosins and defensins, Der p 2, Bet v 1, Ara h 8, Ara h 14, and Ara h 15 (31, 32). One important aspect of both studies is the application of single peanut allergens in the BAT. These were either isolated and purified from raw and in-shell roasted peanuts or recombinantly expressed in *Escherichia coli* (31, 32). The robust and optimized BAT setup using these single allergens allowed the differentiation between peanut-allergic and peanut-sensitized individuals (32).

Mehlich et al. tested alpha-gal sensitized patients in comparison with healthy controls for their basophil reactivity to commercial alpha-gal allergens and pork kidney extract. Thereby, CCR3 was assessed as an identification marker and CD63 as an activation marker for basophils. Similar to the peanut-BAT, they were able to differentiate between patients with alpha-gal syndrome and asymptomatic alpha-gal sensitization within the sensitized patient group using BAT (33).

Chinuki et al. used the BAT to examine the allergenicity of a HWP product in 10 WDEIA patients. The HWP had been produced by acid hydrolysis, but further details on its molecular composition were not provided (5, 34).

We already demonstrated that BAT using CCR3 as identification marker and CD63 as activation marker for basophils allowed the discrimination of patients with WDEIA from controls. ATSs made from peptic hydrolysates of ω5-gliadins, HMW-GS and total gluten showed the best sensitivity and specificity at optimal cut-off (20). Although these three peptic hydrolysates work very well in BAT, one drawback of using those ATSs is that they cannot be easily prepared in routine clinical practice, because the procedure involves elaborate gluten fractionation and digestion (20).

Therefore, we aimed to provide aqueous ATS from gluten samples with different molecular properties that can be easily made for use in BAT. We included four ATSs to cover a wide range of variability in molecular composition. These ATSs were prepared as saline extracts from one representative sample of wheat gluten (G) and of slightly hydrolyzed wheat proteins (sHWPs) and extensively hydrolyzed wheat proteins (eHWPs) selected from our previous work (10). The fourth sample was produced from flour of wheat variety Pamier, a wheat/rye translocation line with an 89% lower content of ω5-gliadins (G-ω5; 2.40 mg/g), the main allergen in WDEIA, in comparison with representative gluten (G; 22.3 mg/g) (35). If G-ω5 truly induced lower allergenic responses, products made of this variety might be nutritionally beneficial for patients with WDEIA. We combined allergenicity assessment using SPT and BAT with the characterization of allergenic proteins in the ATS to identify which proteins are present in those saline ATSs.

## Methods

### Materials

Gluten and HWPs were from Hermann Kröner GmbH (Ibbenbüren, Germany), Tate & Lyle PLC (London, UK), and Manildra Group (Gladesville, Australia). G in the present study corresponds to G1, G-ω5 to G4, sHWP to HWP7, and eHWP to HWP3 (7). All reagents and chemicals were from Sigma Aldrich (Darmstadt, Germany), Merck (Darmstadt, Germany), Carl Roth (Karlsruhe, Germany), Honeywell (Offenbach, Germany), J. T. Baker (Arnhem, The Netherlands), and Fresenius Kabi Deutschland GmbH (Bad Homburg, Germany). Water was purified with an Arium 611VF water purification system (Sartorius, Goettingen, Germany). Pepsin (from porcine mucosa, 10 FIP U/mg), trypsin (from bovine pancreas, TPCK treated, 10.000 BAEE U/mg protein), α-chymotrypsin (from bovine pancreas, TLCK-treated, ≥40 U/mg protein), and thermolysin (from *Geobacillus stearothermophilus*, 30–175 U/mg protein) were purchased from Sigma Aldrich (Darmstadt, Germany) and Merck (Darmstadt, Germany).

### Allergen Test Solutions

To prepare saline ATS for BAT, the sample (eHWP: 25 mg, sHWP: 100 mg, G: 100 mg, and G-ω5: 100 mg) was weighed into a 2 ml tube followed by addition of glass beads for better homogenization and 1 ml 0.9% isotonic NaCl solution. The suspension was homogenized by vortex mixing for 1 min, stirring for 20 min at room temperature, and ultrasonic treatment for 3 min. After centrifugation (2,300 × *g*, 15 min, 20°C), the supernatant was filtered (0.45 μm, regenerated cellulose, GE Healthcare, Chicago, IL, USA) and the protein/peptide concentrations measured at 205 nm by a micro volume UV/VIS spectrophotometer NanoDrop One (Thermo Fisher Scientific, Carlsbad, CA, USA). The ATS from eHWP was diluted 1:5 (v/v) with 0.9% isotonic NaCl solution to adjust protein/peptide concentrations of all ATS for BAT experiments.

Several supernatants of G, G-ω5, and sHWP were prepared, pooled, and lyophilized for ultra-performance liquid chromatography (UPLC)-TripleTOF-MS analysis. The lyophilized powder was carefully homogenized with mortar and pestle and weighed into 2 ml tubes (6 mg). eHWP was used directly (4 mg), because it was completely soluble in isotonic NaCl solution.

### Study Population

Twelve patients with a clinical history of WDEIA based on positive oral food challenge (5 women, 7 men, 26–60 years, median age: 48 years) and 10 individuals without a history of any wheat-related disorder were included in the study as healthy controls (9 women, 1 male, 25–76 years, median age: 44 years). Five of the control subjects were atopic. Further details on the study population are reported in Gabler et al. (20). The study protocol was approved by the ethics committee of the Technical University of Munich and all participants gave written informed consent before being included in the study.

### Skin Prick Test

Skin prick test was carried out on the forearm with gluten (G, G-ω5) and hydrolyzed wheat proteins (eHWPs and sHWPs). Histamine dihypochloride solution (10%) from ALK-Abello (Hørsholm, Denmark) served as a positive control and isotonic NaCl solution from Fresenius Kabi Deutschland GmbH (Bad Homburg, Germany) as a negative control. The SPT was defined as positive, when the wheal diameter caused by the tested substance was ≥3 mm larger than the diameter of the negative control (4).

### Basophil Activation Test

Flow CAST (Bühlmann Laboratories AG, Schönenbuch, Switzerland) was used for quantitative determination of *in vitro* basophil activation, as described previously (20). Anti-FcεRI-mAb and *N*-formyl-methionyl-leucyl-phenylalanine were used as positive controls. Flow cytometry was performed using a FACSCalibur system (Becton-Dickinson Immunocytometry System, Heidelberg, Germany) with a 488 nm, 15 mW and a 635 nm, 10 mW argon laser. Basophils were gated as low side scatter CCR3/side scatter^low^. CCR3 was used as identification marker for basophils and CD63 as basophil activation marker, labeled with anti-CCR3-phycoerythrin mAb and anti-CD63-fluorescein-isothiocyanate, respectively. BD CellQuest (Becton-Dickinson Immunocytometry System) was used to analyze the data. At least 450 basophils were counted per measurement (13, 28). The following BAT parameters were studied: basophil activation (%CD63^+^ basophils) expressed as percentage of basophil granulocytes expressing CD63 divided by the total number of counted basophil granulocytes per single measurement and %CD63^+^ basophils/anti-FcεRI ratio as quotient of the basophil activation (%CD63^+^ basophils) triggered by ATS and by the anti-FcεRI mAb as positive control (33).

### Sodium Dodecyl-Sulfate Polyacrylamide Gel Electrophoresis

Sodium dodecyl-sulfate polyacrylamide gel electrophoresis was carried out according to Lagrain et al. (36). In brief, lyophilized ATS (G, G-ω5, sHWP) and eHWP (used directly, because of complete solubility in isotonic NaCl) were incubated with reducing extraction buffer for 12 h at room temperature, heated to 60°C for 10 min and centrifuged (5,000 × *g*, 20°C, 5 min). A homogeneous NuPAGE 10% polyacrylamide Bis-Tris gel (10 mm × 1 mm wells) (Invitrogen, Carlsbad, CA, USA) was used with a MOPS running buffer. The PageRuler Unstained Protein Ladder served as a molecular mass (M_r_) standard (Thermo Fisher Scientific). The running time was 30 min at 200 V and 115 mA. Protein bands on the gel were fixed with 12% trichloroacetic acid (w/w) (30 min), stained with Coomassie blue (30 min) and destained in two steps. The gels were scanned using the Gel Doc EZ Imager (Bio-Rad Laboratories, Munich, Germany) and the Image Lab software (Bio-Rad Laboratories) (10, 37).

### Gel Permeation HPLC

Two different gel permeation (GP)-HPLC systems, previously reported by Gabler at al. and Scherf et al. were used to analyze the M_r_ distribution of proteins and peptides in the ATS compared with protein markers of known M_r_ (10, 38). Measurements were performed on a Jasco HPLC Extrema (Jasco, Gross-Umstadt, Germany). A BioSep-SEC-s3000 column (300 mm × 4.6 mm, 29 nm, 5 μm, Phenomenex, Aschaffenburg, Germany) was used for protein separation with an isocratic gradient (50:50, 0.1% trifluoroacetic acid (TFA) in ultrapure water/0.1% TFA in acetonitrile) with a flow rate of 0.3 ml/min at 20°C. Chromatography was carried out on a BioBasic SEC-60 column (150 mm × 7.8 mm, 6 nm, 5 μm, Thermo Fisher Scientific) with an isocratic gradient (70:30, 0.1% TFA in ultrapure water/0.1% TFA in acetonitrile) at a flow rate of 1.0 ml/min for small proteins/peptides. The injection volume was 3–5 μl.

### Reversed-Phase HPLC

The protein/peptide concentration of the ATS was analyzed according to Gabler et al. using reversed phase (RP-)HPLC on a Jasco XLC instrument (Jasco) using a C_18_ column at 60°C (Acclaim 300, C_18_, 2.1 mm × 150 mm, 300 nm, 3 μm, Thermo Fisher Scientific). The elution solvents were 0.1% TFA in ultrapure water (A) and 0.1% TFA in acetonitrile (B) at a flow rate of 0.2 ml/min. Gradient elution was performed: 0 min 0% B, 0.1–0.5 min 24% B, 0.6–15 min 56% B, 15.1–19.1 min 90% B, 19.2–35.0 min 0% B. The injection volume was 20 μl. Prolamin Working Group (PWG)-gliadin was used for external calibration (10, 39).

### Ultra-Performance Liquid Chromatography (UPLC)-TripleTOF-MS

#### Reduction and Alkylation

Lyophilized ATS from G, G-ω5, and sHWP as well as eHWP were dissolved in 320 μl of TRIS-HCl buffer (0.5 mol/L, pH 8.5) and 320 μl 1-propanol. For reduction, 50 μl of Tris-(2-carboxyethyl)-phosphine (TCEP) solution (22 mg/ml TCEP in TRIS-HCl buffer) were added and the samples shaken for 30 min at 60°C under nitrogen. After cooling, 100 μl of chloroacetamide (CAA)-solution was added for alkylation (34 mg/ml CAA in TRIS-HCl buffer). The samples were shaken for 45 min at 37°C in the dark. The solutions were evaporated to dryness (37, 40).

#### Enzymatic Digestion

Different protein digestions were carried out: pepsin + trypsin (PT), pepsin + chymotrypsin (PC), pepsin + trypsin + chymotrypsin (PTC), trypsin + chymotrypsin (TC), and thermolysin (TLY). Digestion was performed by adding pepsin [750 μl, 0.2 mg/ml in 0.15 mol/L HCl, pH 2, enzyme/substrate (E:S) ratio of 1:20 (w/w)] to the alkylated residues and shaking for 60 min at 37°C. After the peptic digest, the pH was adjusted to 6.5 with PBS (50 mmol/L). Then, trypsin and/or chymotrypsin [E:S of 1:20 for T or C, E:S of 1:40 for TC (w/w)] were added and the samples were hydrolyzed for 120 min at 37°C. For TC digestion, TC was added to the alkylated residues [1 ml, 0.12 mg/ml T/C in 0.1 mol/L TRIS-HCl-buffer, E:S of 1:50 (w/w)] followed by incubation for 16 h at 37°C. The digestions were stopped by heating for 10 min at 95°C (37, 40). TLY digestion [E:S of 1:20 (w/w)] was carried out in TRIS-HCl CaCl_2_ buffer (0.2 mol/L TRIS, 0.5 mmol/L CaCl_2_· 2H_2_O, pH 6.5) at 37°C for 16 h. The reaction was stopped with formic acid (FA) (41–43).

#### Solid Phase Extraction

Enzymatic digests were purified by solid phase extraction (SPE) using 100 mg Discovery DSC-18 cartridges (Supelco, Bellefonte, PA, USA). After activation with methanol, equilibration with 80/20 (v/v) acetonitrile 0.1% FA in water and washing with 2/98 (v/v) acetonitrile/0.1% FA the cartridges were loaded with sample and washed again. Elution was carried out using 40/60 (v/v) acetonitrile/0.1% FA in the first step and 80/20 (v/v) acetonitrile/0.1% FA in the second. Both eluates were united and evaporated to dryness. The residues were dissolved in 500 μl 0.1% FA and filtered immediately before UPLC-TripleTOF-MS analysis (40, 44).

#### UPLC-TripleTOF-MS

The UPLC-TripleTOF-MS analysis was performed using an UPLC system ExionLC coupled to a TripleTOF 6600 MS (SCIEX, Darmstadt, Germany). A bioZen peptide PS-C18 column (100 mm × 2.1 mm, 10 nm, 1.6 μm) (Phenomenex) was used. Peptides (injection volume 10 μl) were separated using linear gradient elution (0–65 min 5% B to 100% B, 65–69 min 100% B, 69–70 min 100% B to 5% B, 70–75 min 5% B; solvent A: 0.1% FA in water, solvent B: 0.1% FA in acetonitrile) with a flow rate of 0.35 ml/min at 40°C. The MS was operated in positive electrospray ionization mode and the following settings: ion spray voltage 5,500 eV, source temperature 550°C, heating gas 0.45 MPa, nebulizing gas 0.38 MPa, curtain gas 0.24 MPa.

The MS was operated in information-dependent acquisition (IDA) mode. The mass-to-charge range for MS1 was 350–1,800, using an accumulation time of 250 ms, collision energy of 10 V, and a declustering potential of 80 V. The IDA criteria for the precursor ion included intensity of >100 counts/s and the resolution was set to 0.5 Da. MS2 spectra of the 20 most abundant compounds were recorded in a mass-to-charge range of 350–1,800, using an accumulation time of 40 ms, collision energy of 35 V, declustering potential of 80 V, and a collision energy spread of 5 V. Instrument control and data acquisition were performed with Analyst TF software (v 1.7.1., SCIEX).

#### Analysis of UPLC-TripleTOF-MS Data

The raw data were analyzed against the proteome of *Triticum aestivum* (UniprotKB, download 08/2019) using the proteomics software MaxQuant (version 1.6.3.4) (45). The search parameters including specific and unspecific digestion are reported in Supplementary Table 1. All other parameters were kept as default settings. The intensity based absolute quantitation (iBAQ) algorithm implemented in MaxQuant was used to estimate wheat protein abundances in the ATS. A total sum normalization of protein iBAQ intensities between sample measurements was performed to correct for different total protein injection amounts (37, 40).

### Statistical Analysis

A statistical analysis was performed with Origin 2020 (OriginLab Cooperation, Northampton, MA, USA) and SigmaPlot 14 (Systat Software GmbH, Erkrath, Germany). One-way ANOVA with Dunn's *post-hoc* test (*p* < 0.05) was used to identify significant differences between the ATS analyzed by HPLC, SPT, and BAT. Receiver operating characteristic (ROC) analyses were carried out to estimate how well BAT parameters, such as area under the ROC curve (AUC) distinguished between patients and controls. The optimized discrimination threshold (cut-off) for the %CD63^+^ basophils/anti-FcεRI ratio was determined based on the ROC curve for best selectivity and specificity.

## Results

### Allergenicity of Gluten and HWP for Patients With WDEIA

#### Skin Prick Test

**Figure 1:**
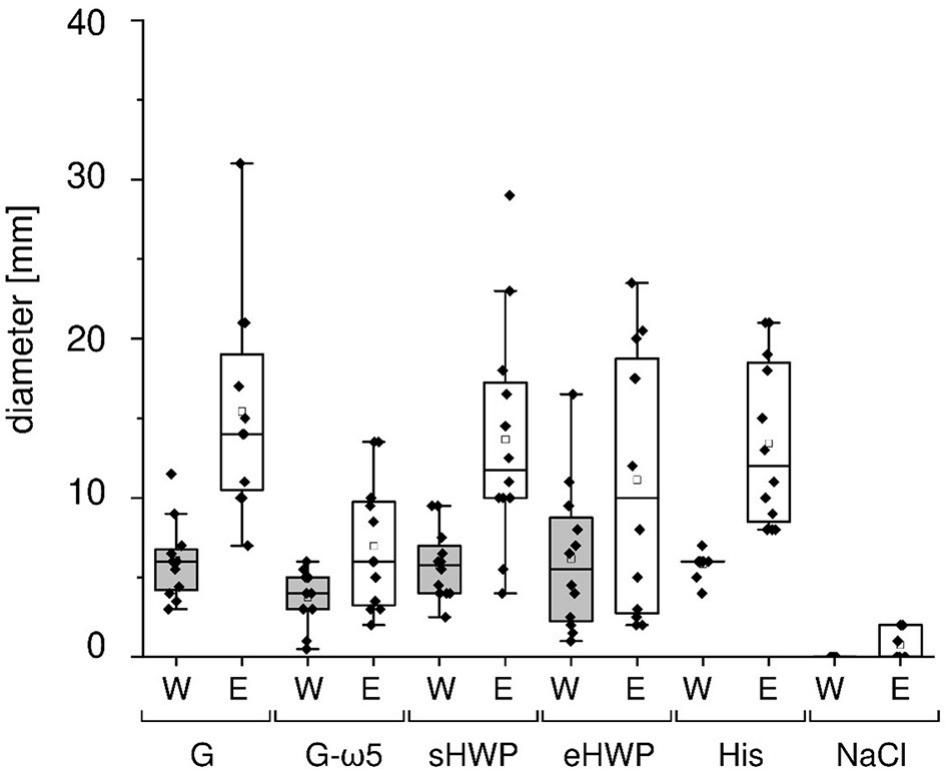
Skin prick test results of patients with wheat-dependent exercise-induced anaphylaxis. Allergen test solutions from gluten G and G-ω5 and from slightly and extensively hydrolyzed wheat proteins sHWP and eHWP were used, as well as histamine dihypochloride (10%) solution (His) as positive control and isotonic sodium chloride solution as negative control (NaCl). The diameter of the wheals (W) and erythema (E) were documented in mm. A double determination was performed for each patient (*n* = 2), except patient 4 (*n* = 1). The data for gluten G were added for comparison and were already reported in Gabler et al. (20).

As expected, all patients with WDEIA showed sensitizations to the positive control (wheal and erythema mean diameter (W/E): 5.8 and 13.4 mm), but none to the negative control (0 mm). A positive reaction was triggered in all patients with WDEIA for G (W/E 6.1 and 15.4 mm), in 11 of 12 patients for sHWP (W/E 5.8 and 13.7 mm), in 10 of 12 patients for G-ω5 (W/E 3.8 and 7.0 mm), and in 9 of 12 patients for eHWP (W/E 6.2 and 11.1 mm) (Figure 1 and Supplementary Table 2). Large interindividual differences were observed that resulted in wide ranges of minimal and maximal diameter for each substance, ranging from 0.5 to 16.5 mm for W and from 2.0 to 31.0 mm for E overall. There were no significant differences (*p* > 0.05) in mean wheal diameter between the four substances, even if G, sHWP, and eHWP triggered wheals that were comparable in size with those of the positive control and about 60% larger compared with G-ω5. The mean erythema diameter caused by G, sHWP, and eHWP was also similar to that of the positive control. The erythema following SPT with G was significantly higher (*p* < 0.05) than that with G-ω5, but all other pairwise comparisons were not significantly different from one another.

#### Basophil Activation

**Figure 2:**
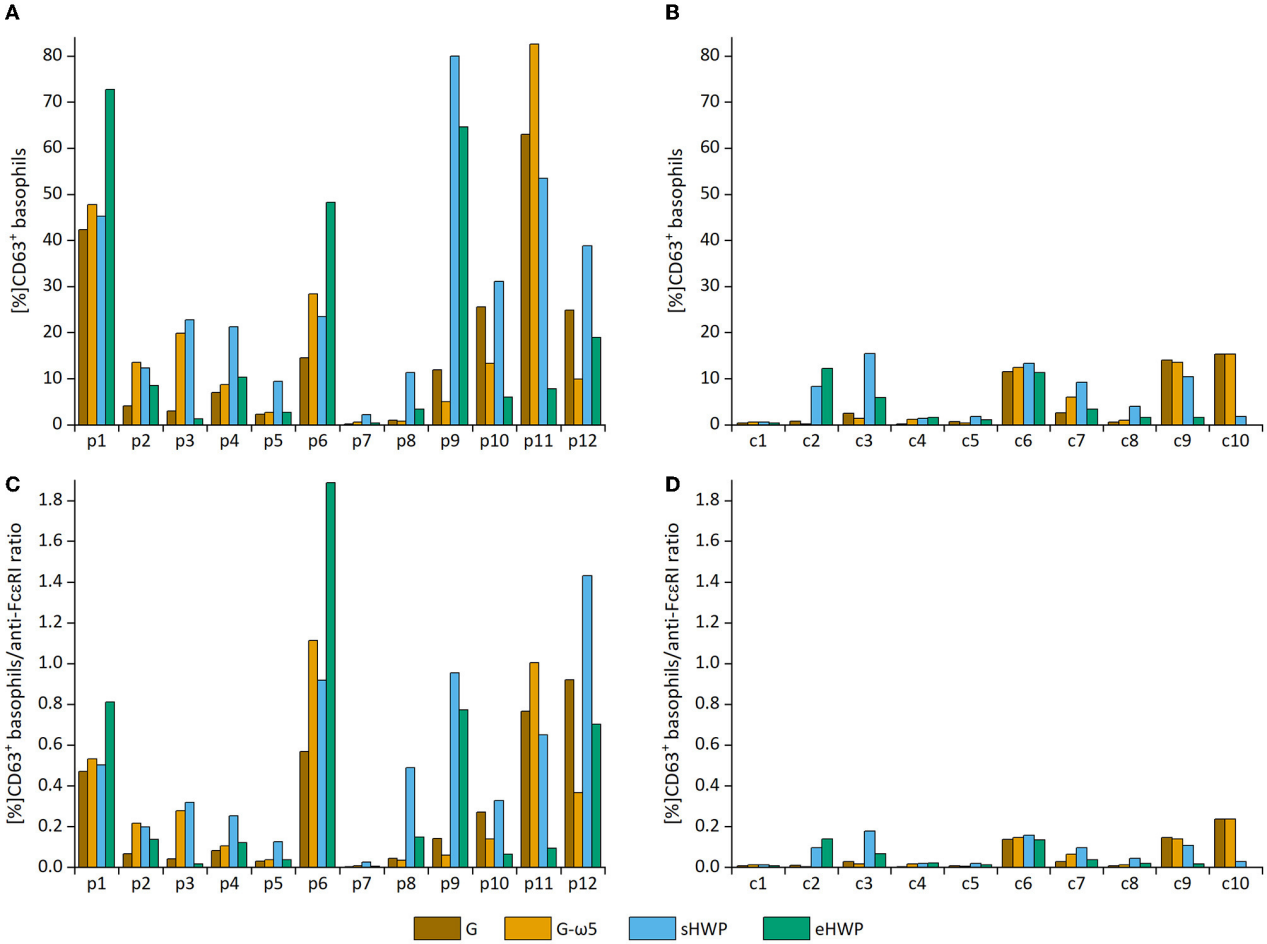
Basophil activation in individual patients and controls. Allergen test solutions from gluten G and G-ω5 and from slightly hydrolyzed wheat proteins (sHWPs) and extensively hydrolyzed wheat proteins (eHWP) were used. **(A)** %CD63^+^ basophils from patients p1–p12, **(B)** %CD63^+^ basophils from controls c1–c10, **(C)** %CD63^+^ basophils/anti-FcεRI ratio from patients p1–p12, **(D)** %CD63^+^ basophils/anti-FcεRI ratio from controls c1–c10.

**Figure 3:**
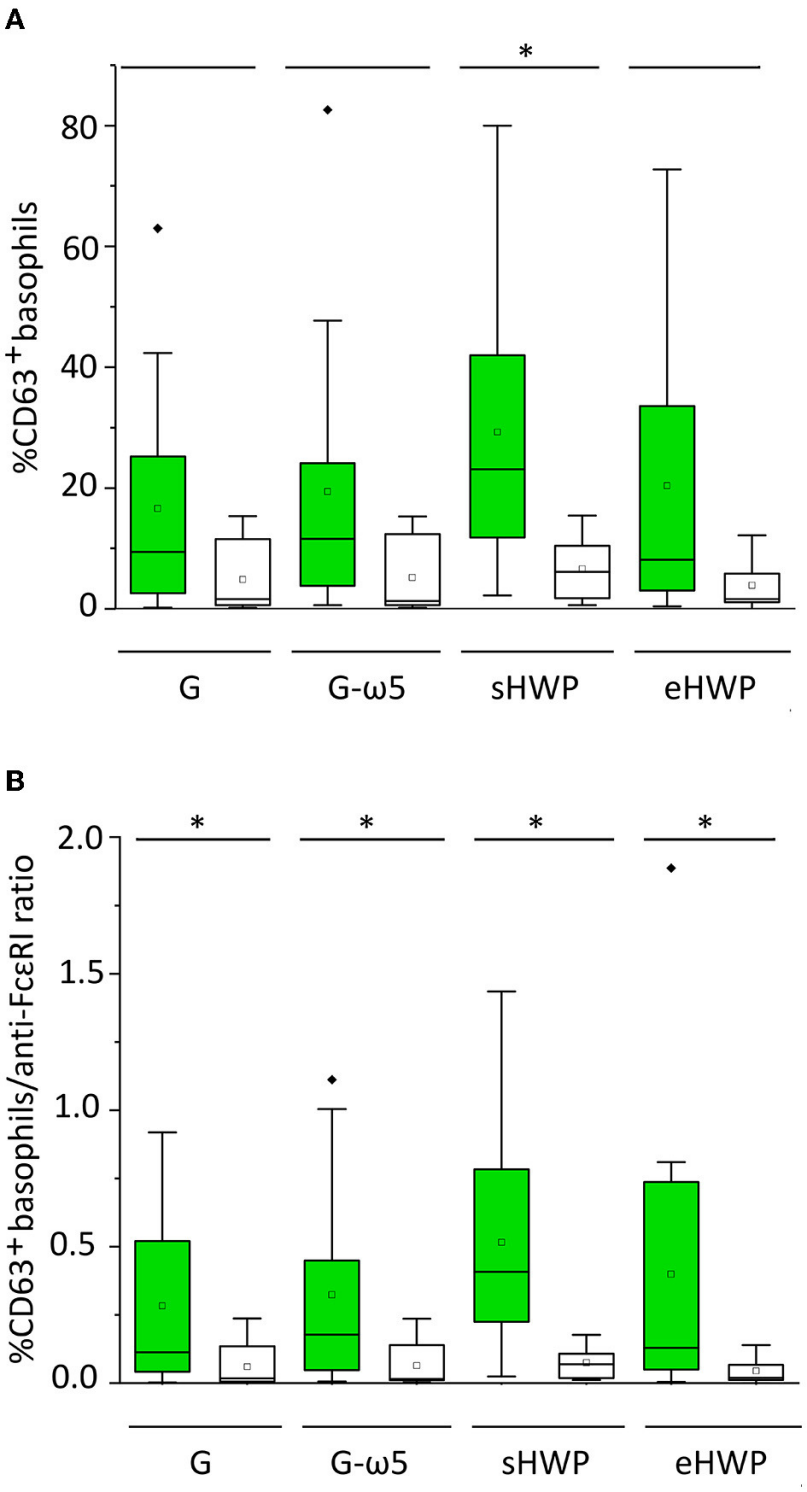
Basophil activation in patients (green) and controls (white). Allergen test solutions from gluten G and G-ω5 and from sHWPs and eHWPs were used. **(A)** %CD63^+^ basophils, **(B)** %CD63^+^ basophils/anti-FcεRI ratio. Significant differences between patients and controls are indicated by asterisks (one-way ANOVA, Dunn's *post-hoc* test, *p* < 0.05). There were no significant differences between patients' reactivity to different ATS. Diamonds indicate individual outliers outside the first or third quartiles. Squares represent the mean and lines the median. Whiskers mark the 1.5 interquartile range. The box corresponds to the range in which the middle 50% of the data are located.

All ATS for gluten and HWP induced basophil activation in the blood of patients with WDEIA, except for p5, p7, and p8 (Figure 2 and Supplementary Figures 1–8). As already observed in the SPT, the responses were highly individual, e.g., with blood from patients p1 and p6 showing the highest basophil activation for eHWP, p9 for sHWP and eHWP, and p11 for G and G-ω5. Contrary to expectations, G-ω5 did not lead to lower basophil activation in comparison with G in general. The basophil activations (%CD63^+^ basophils) of patients were in a range between 0.2 and 63.0% (median: 9.4%) for G, 0.6–82.6% (median: 11.6%) for G-ω5, 0.4–72.7% (median: 8.2%) for eHWP, and 2.2–80.0% (median: 23.1%) for sHWP. Significant differences in %CD63^+^ basophils between patients and controls were found for sHWP (*p* < 0.05), but not for G, G-ω5, and eHWP. In contrast, patients showed significantly higher %CD63^+^ basophils/anti-FcεRI ratios compared with controls with all ATS (*p* < 0.05) (Figure 3). Consequently, the %CD63^+^ basophils/anti-FcεRI ratio was used as characteristic parameter for further investigations.

There were no significant differences (*p* > 0.05) in patient %CD63^+^ basophils/anti-FcεRI ratios between the four different ATS (median G = 0.113, G-ω5 = 0.178, eHWP = 0.130, and sHWP = 0.408), *inter alia*, due to high interindividual variability. The %CD63^+^ basophils/anti-FcεRI ratios were low for all ATS in controls (median: G: 0.018, G-ω5: 0.016, eHWP: 0.069, and sHWP: 0.019). The ROC curves generated for all ATS from the %CD63^+^basophils/anti-FcεRI ratio of patients and controls revealed that BAT with sHWP gave the highest AUC (0.925) with excellent sensitivity (100%) and specificity (83%) to discriminate between patients with WDEIA and controls (Supplementary Figure 9 and Supplementary Table 3).

### Identification of Allergenic Proteins in the Test Solutions

#### Sodium Dodecyl-Sulfate Polyacrylamide Gel Electrophoresis

**Figure 4:**
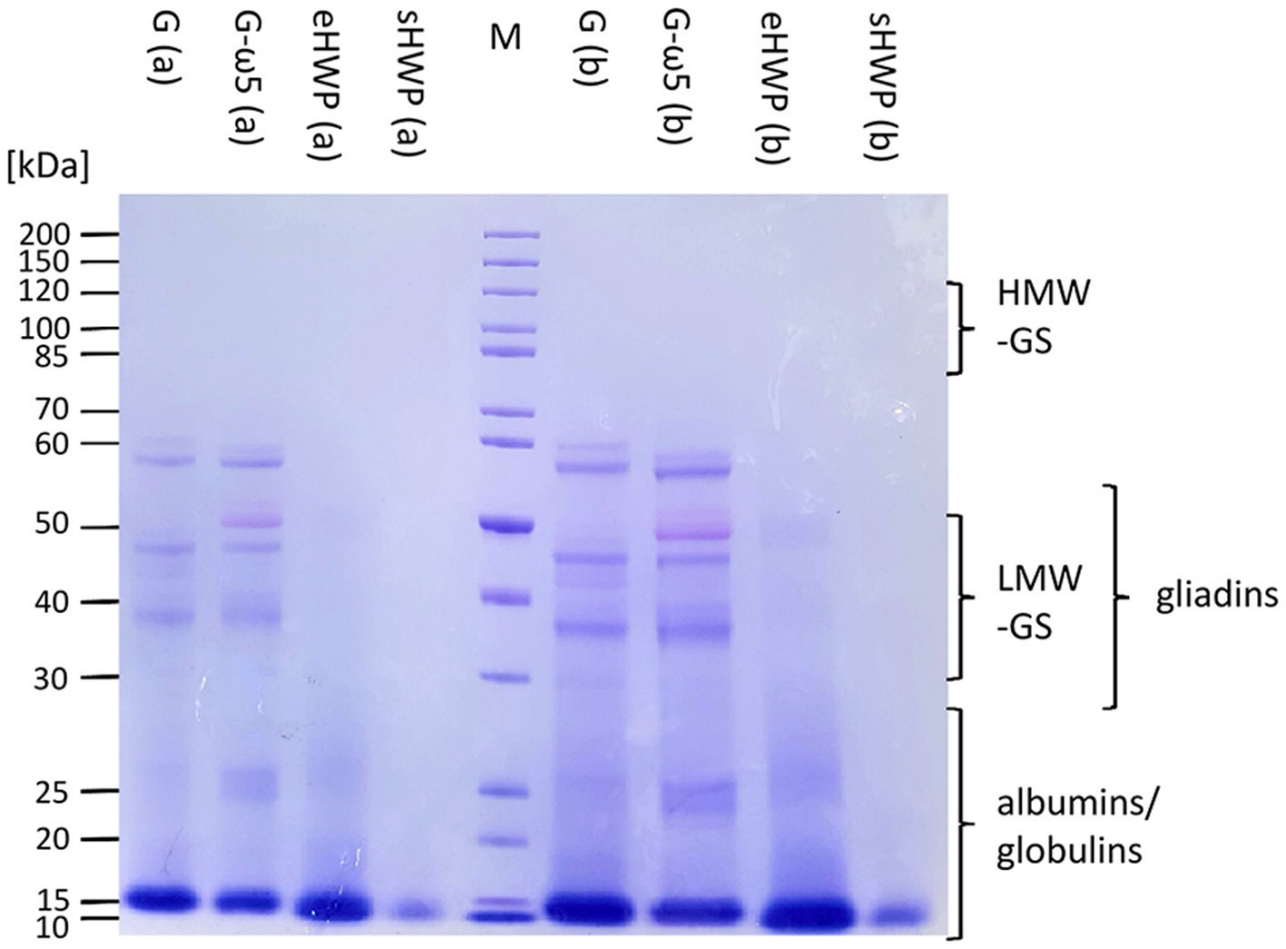
Sodium dodecyl-sulfate polyacrylamide gel electrophoresis (SDS-PAGE) of allergen test solutions. Gluten G and G-ω5 as well as sHWPs and eHWPs were analyzed. Protein marker (M) 3.5 μg, samples 5.3 μg (a), and 15.0 μg (b). HMW-GS, high-molecular-weight glutenin subunits, LMW-GS, low-molecular-weight glutenin subunits. The albumin/globulin fraction may consist of, e.g., grain softness proteins, puroindolines, purothionins (Tri a 37), non-specific lipid-transfer protein (Tri a 14), and amylase/trypsin-inhibitors (Tri a 15, Tri a 28, Tri a 29, Tri a 30, and Tri a 40) (40).

In SDS-PAGE, all protein bands from the ATS had M_r_ below or equal to 60 kDa (Figure 4). The lack of larger and hydrophobic proteins, such as HMW-GS was expected, because the ATSs were aqueous extracts of G, G-ω5, and sHWP or were completely soluble in water as in case of eHWP. The band pattern of G and G-ω5 was similar with bands at 60, 57, 47, and 37 kDa and three additional ones at 52, 40, and 27–24 kDa for G-ω5. Bands with M_r_ about 60 kDa typically belong to ω-gliadins and the additional band at 52 kDa in G-ω5 is likely to be from ω-secalins. The other bands in the range from 37 to 47 kDa can be assigned to gliadins and LMW-GS (46). While eHWP showed a weak and blurred band at 20–27 kDa and its main band at 10–16 kDa, sHWP had only one band at 10–16 kDa. This indicates that proteins were degraded through hydrolysis in sHWP and eHWP. The most intense protein band in all ATSs was at M_r_ 10–16 kDa and this range corresponds to non-gluten proteins of the water-/salt-soluble albumin/globulin fraction, such as grain softness proteins, puroindolines, purothionins (Tri a 37), non-specific lipid-transfer protein (Tri a 14), and ATIs (Tri a 15, Tri a 28, Tri a 29, Tri a 30, and Tri a 40), many of them already known as allergens (47).

#### High-Performance Liquid Chromatography

**Figure 5:**
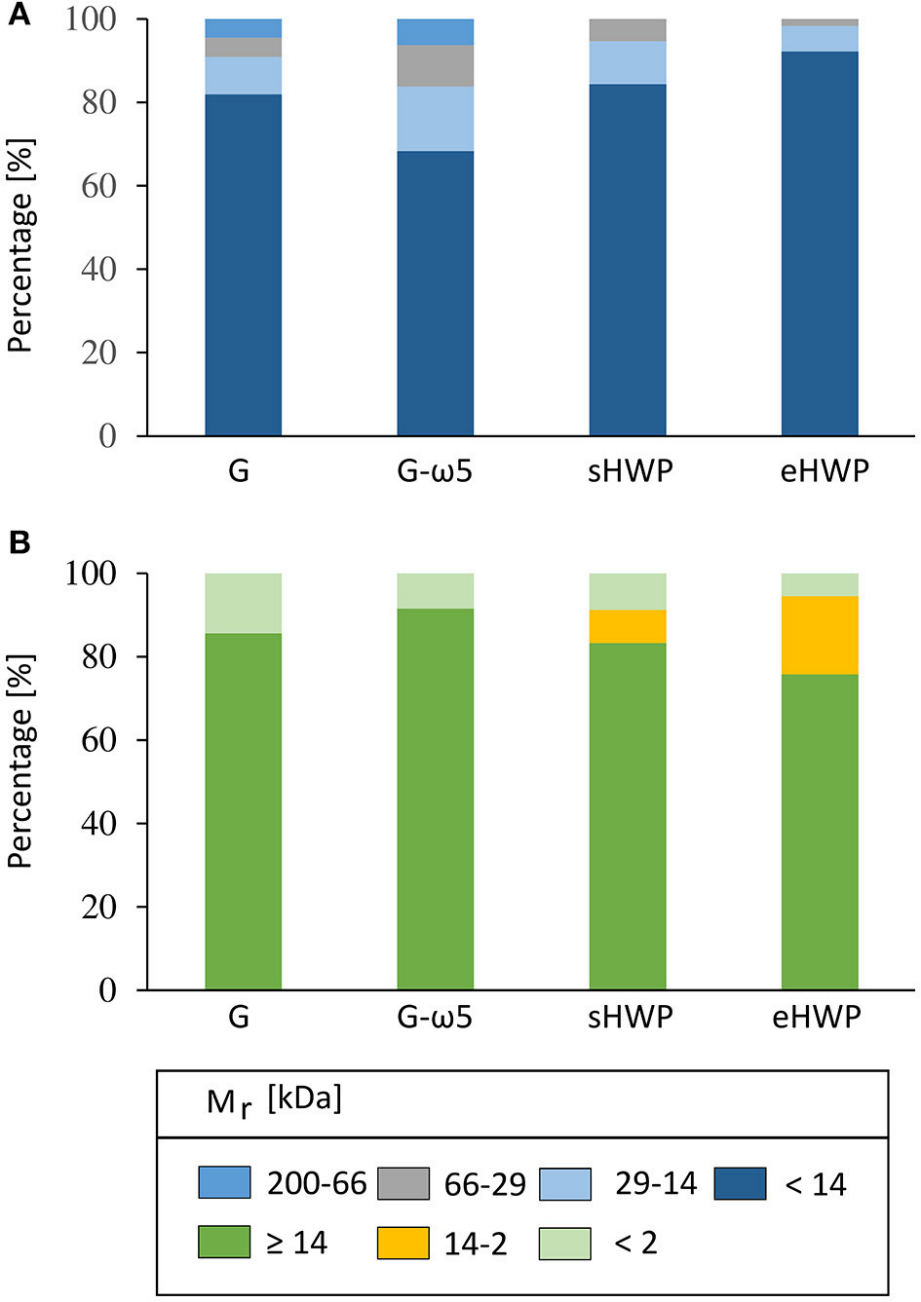
Gel-permeation high-performance liquid chromatography (GP-HPLC). Relative molecular mass distribution (M_r_) [%] of the allergen test solutions from gluten G and G-ω5 and from sHWPs and eHWPs. Areas within each fraction were set by marker substances. Two different systems were used. **(A)** 1: 200–66 kDa; 2: 66–29 kDa; 3: 29–14 kDa; 4: <14 kDa. GP-HPLC; **(B)** 1: ≥14 kDa; 2: 14–2 kDa; 3: <2 kDa.

Gel permeation- and RP-HPLC analyses were carried out to obtain further information complementary to SDS-PAGE on the M_r_ distribution of the proteins in the ATS and their hydrophobicity profile. Both GP-HPLC systems showed that there were high percentages of proteins with M_r_ of about 14 kDa present in the ATS (Figure 5). More than 68% of all proteins in the four ATS had a M_r_ about or below 14 kDa, according to system I suitable for a M_r_ range from <14 to 200 kDa (G: 81.9%, G-ω5: 68.3%, sHWP: 84.4%, and eHWP: 92.2%) (Supplementary Figure 10). System II suitable for a M_r_ range from <2 to ≥ 14 kDa confirmed that over 75% of proteins in the ATS had a M_r_ about 14 kDa (G: 85.6%, G-ω5: 91.5%, sHWP: 83.3%, and eHWP: 75.7%) (Supplementary Figure 11). These results corresponded well to the protein band pattern on the SDS-PAGE gel.

The RP-HPLC chromatograms of the G and G-ω5 ATS showed the typical hydrophobicity profile of the albumin/globulin fraction. In contrast, the peaks in the chromatograms of sHWP and eHWP could not be clearly assigned to any reference chromatogram of intact wheat proteins, again indicating protein degradation (Supplementary Figure 12). The protein concentrations of the ATS used for the BAT experiments determined by RP-HPLC were 2.10 mg/ml (G), 2.05 mg/ml (G-ω5), 3.96 mg/ml (sHWP), and 3.00 mg/ml (eHWP). Higher protein concentrations were not achievable with this preparation procedure for G, G-ω5, and sHWP, because of limited solubility. The concentration range of the four ATS, in which the allergenic basophil activation was triggered, was not directly comparable between the ATS. The concentrations were not set in a specific range, but resulted from preliminary tests, which were primarily intended to exclude non-specific activations in the control group while triggering specific activations in patients.

### Proteomics-Based Untargeted Liquid-Chromatography Mass Spectrometry of the ATS

**Figure 6:**
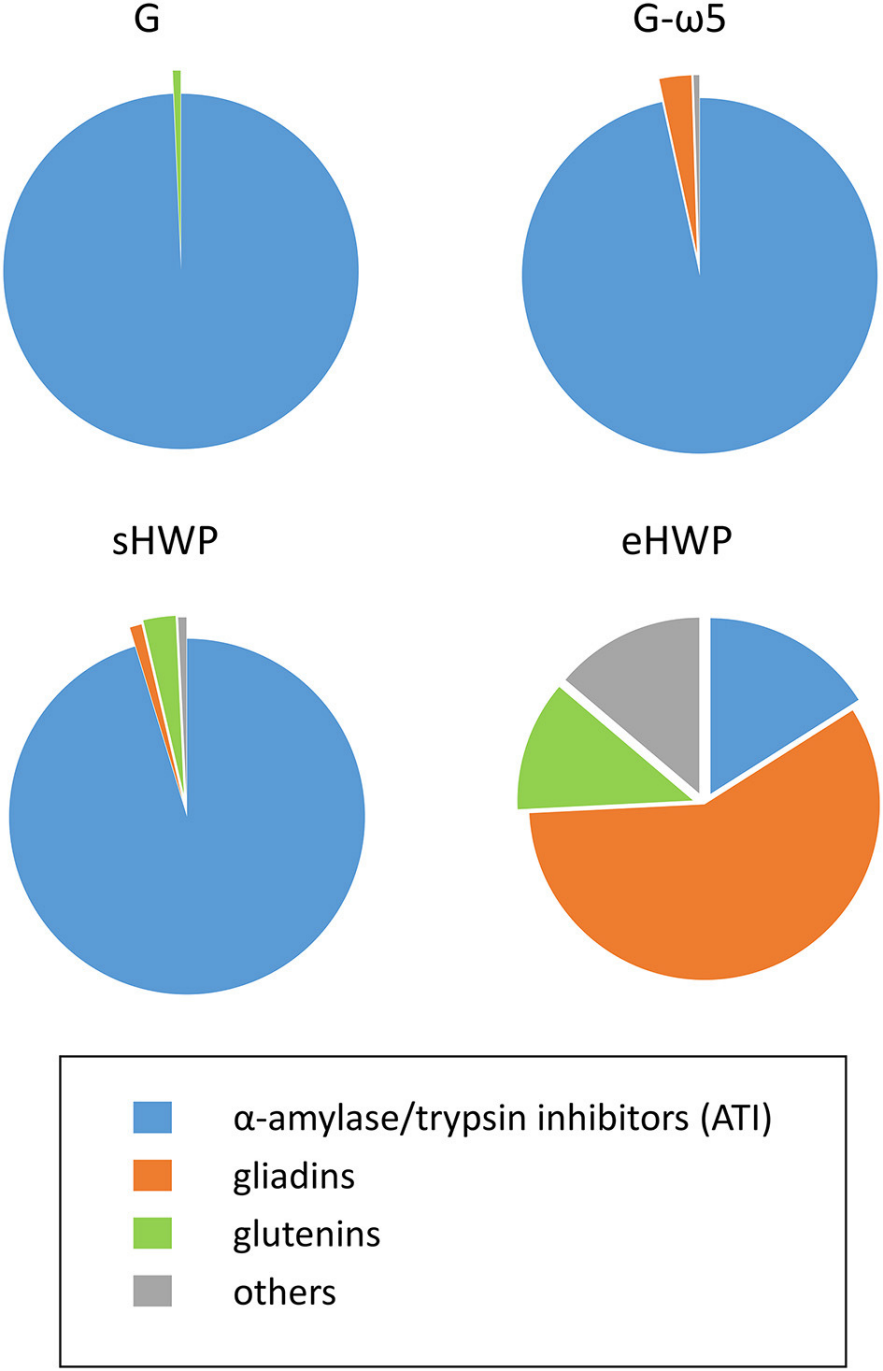
Percentages of protein groups in allergen test solutions. Groups include α-amylase/trypsin inhibitors (ATI, blue), gliadins (orange), glutenins (green), and others (gray) in allergen test solutions from gluten G and G-ω5 and from sHWPs and eHWPs. Percentages were determined using the intensity based absolute quantitation (iBAQ) algorithm with ultra-performance liquid chromatography (UPLC)-TripleTOF-MS data (protein score > 40) in specific digestion mode (pepsin + trypsin). Details on specific proteins are available in Supplementary Tables 4–11.

While SDS-PAGE and HPLC already provided valuable information on the identities of the proteins in the ATS, untargeted UPLC-TripleTOF-MS of different enzymatic digests of the ATS was performed to identify the specific proteins in the ATS and their proportions. Different enzyme combinations were used to maximize protein identifications and avoid bias, because gluten proteins, and especially ω5-gliadins, are known to be resistant to cleavage with P, T, or C (40). Of the PT, TC, PTC, PC, and TLY digestions used (Figure 6 and Supplementary Figure 13), PT turned out to be the most suitable, because percentages of identified proteins in the ATS were the highest in comparison with other digestions. Consequently, the peptides and corresponding proteins identified in the ATS after PT digestion are reported in Supplementary Tables 4–11, using both specific and unspecific digestion mode for data evaluation.

The identified proteins in the ATS made from gluten samples G and G-ω5 contained 96.6 and 99.3% of ATIs, such as ATI-types CM1, CM2, CM3, CM16, 0.28, and 0.53 in G and CM2, CM3, CM16, and 0.19 in G-ω5. ATIs are soluble in aqueous salt solutions whereas gluten, by definition, remains mostly insoluble. Therefore, it appears reasonable that ATIs were enriched during ATS preparation with isotonic NaCl solution. Small proportions of LMW-GS and α-gliadins were present in G, as well as α- and ω-gliadins in G-ω5.

The solubility of sHWP in aqueous solutions was comparable with that of gluten samples (10) as was the composition of the ATS. It consisted of 88.8% of ATIs, such as ATI-types CM1, CM2, CM3, CM16, 0.19, and 0.28, as well as a slightly higher proportion of 3.7% of gluten proteins (LMW-GS, gliadins) compared with G and G-ω5. In contrast, eHWP contained 70.2% of gluten proteins, with 58.2% gliadins (α-, β-, γ-gliadins) and 12.0% glutenins (LMW-GS). This difference can be explained by the fact that eHWP was strongly hydrolyzed and completely soluble in aqueous solutions. ATIs (CM1 and CM3) only represented 16.0% of proteins in eHWP and the remaining 13.8% were other proteins, such as enzymes and uncharacterized proteins. The investigations using TC, PTC, PC, and TLY digestions showed some variation in protein composition compared with the PT digestion, but the overall picture of identified protein groups in the ATS was similar (Supplementary Figure 13).

The sequences of the identified proteins in the ATS were analyzed for known WDEIA epitopes (15, 48, 49). Only the epitope QQPGQ was identified two times in an ω-gliadin (Uniprot accession: C0KEH9) present in G-ω5. All other identified proteins in the ATS contained none of the known WDEIA epitopes.

## Discussion

We expected to see differences in allergenicity to patients with WDEIA between G and G-ω5, because G-ω5 was gluten from a wheat/rye-translocation line (35) that contains a significantly lower amount of ω5-gliadins. SPT results showed that wheal and erythema diameters caused by G-ω5 were the lowest compared with other substances, but the differences were not significant except for the comparison of erythema diameter between G and G-ω5. However, the BAT %CD63^+^ basophils/anti-FcεRI ratio was similar for G and G-ω5 with almost identical median values and ranges, as were all parameters derived from the ROC curves. It was reasonable to assume that gluten with a significantly lower ω5-gliadin content would trigger lower basophil activations in patients than representative gluten, since ω5-gliadins are considered to be the main allergen of WDEIA (14). In several cases, it appeared as if even the opposite was the case, because stronger basophil activities occurred for G-ω5 in comparison to G in p1, p2, p3, p4, p6, and p11. These results indicate that other allergenic proteins need to be relevant and present in the ATS.

Altenbach et al. used transgenic wheat with reduced content of ω5-gliadins and assessed its allergenicity in sera of eleven patients with WDEIA using a two-dimensional immunoblot analysis. Seven out of eleven patients showed reduced levels of immunoglobulin E (IgE) reactivity to ω5-gliadins using transgenic wheat, but the same sera showed IgE reactivities to other gluten proteins at the same time. Additionally, sera from three patients generally had the highest IgE reactivity not to ω5-gliadins, but to HMW-GS, α-gliadins, and non-gluten proteins. They concluded that this transgenic wheat line was not beneficial for the nutrition of patients with WDEIA because of the complexity of the immune response in the participating patients with WDEIA. Without knowing to which wheat protein groups, a patient with WDEIA is sensitized, it is too risky overall to consume transgenic wheat. Even if the ω5-gliadin content is reduced therein, other wheat proteins were shown to trigger IgE reactivity in patients with WDEIA (50). Our findings support their conclusion and still leave a wheat-free diet and/or avoidance of cofactors as the only safe option for patients with WDEIA. Further, the identified proteins in ATS from G and G-ω5 both contained over 96% of ATIs (non-gluten proteins) and only very low proportions of gliadins, so that a potential difference in ω5-gliadin content was most likely negligible.

We expected to find LTPs in the aqueous ATS, as they are known to be soluble and to cause basophil activity in patients. Pastorello et al. described three cases of exercise-food challenge confirmed patients with WDEIA. They identified a 9 kDa LTP as the allergenic protein in these patients by immunoblotting. Simultaneously, these patients showed no reactivity to the gliadin and glutenin fractions (23). The protein band of the albumin/globulin fraction (10–15 kDa) of G-ω5 and sHWP in the SDS-PAGE gel of the lyophilized ATS suggested that LTPs may be present. However, no LTPs were identified with the proteomics UPLC-TripleTOF-MS approach (PT-digestion), but high percentages of ATIs. In our previous study, the same WDEIA patient cohort was tested for sIgE against LTP. All patients showed negative results (<0.1 KU/L; LTP/Tri a 14) (20).

Based on the heterogeneous molecular properties of sHWP and eHWP, we expected differences among the parameters investigated, but we did not find any significant differences in SPT or CD63^+^ basophils/anti-FcεRI ratio. The only parameters that differed were those derived from the ROC curves indicating that sHWP yielded higher sensitivity/specificity (100%/83%) compared with eHWP (75%/70%) to discriminate between patients and controls. Due to a lack of studies so far, it remains unclear how degree and type of protein hydrolysis affect the allergenicity of gluten in WDEIA. Hydrolysis to a certain degree may increase the allergenicity by exposing epitopes or generating new ones (6, 7, 51). Beyond that degree, continued hydrolysis is expected to decrease allergenicity, because epitopes are degraded.

Neither SPT nor BAT revealed clear differences between gluten samples (G, G-ω5) and HWP (sHWP, eHWP) in terms of allergenicity. SDS-PAGE and GP-HPLC revealed that all ATS contained high percentages of proteins with M_r_ 10–16 kDa (SDS-PAGE) and about 14 kDa (GP-HPLC). UPLC-TripleTOF-MS analysis showed that high percentages of ATIs were present and their M_r_ correspond exactly to this mass range. These findings raise the question, whether ATIs are implicated not only in baker's asthma, but also in WDEIA. Until now, the main focus was on gluten proteins, such as ω5-gliadins and HMW-GS as major WDEIA allergens (19, 48, 52), though there are reports that ATIs may also play a role in WDEIA (24). IgE immunoblotting with patients' sera showed reactions to ATIs present in wheat protein fractions and ATI-types CM1, CM3, CM16, and 0.19 were identified in the allergenic fraction (24), similar to our results.

The sequences of all identified proteins in the ATS were analyzed for known WDEIA epitopes (15, 48, 49). Only one known epitope (QQPGQ) was identified, indicating that other epitopes appear to be relevant in WDEIA which are currently unknown. Western blotting using patient samples can be used in further studies to support their identification.

Sandiford et al. and Battais et al. reported possible cross-reactive epitopes between gliadins and ATIs (53, 54). Pastorello et al. used wheat flour within their study, which naturally contains ATIs, whereas we used gluten. By definition, gluten is poorly soluble in water and salt solutions, but residues of the soluble albumin/globulin fraction still remain in the gluten polymer, even after extensive washing to remove starch and other flour constituents (38, 55).

Besides ATIs, small percentages of gliadins and/or LMW-GS were present in the ATS from G, G-ω5, and sHWP. Overall, these three ATS showed a high degree of similarity regarding protein composition and solubility, again confirming that sHWP was only slightly hydrolyzed.

The basophil activation triggered by eHWP was according to expectations, because over 70% of proteins were gliadins and glutenins, but rendered soluble due to more extensive hydrolysis compared with sHWP. As gluten proteins are already known as relevant allergens in WDEIA, basophil activation in patients was anticipated (4, 48). Chinuki et al. reported a HWP product in soap, which was produced by acid hydrolysis and triggered allergic reactions in patients with WDEIA (5, 19). Apparently the degree of hydrolysis was enough to solubilize all wheat proteins in water, but not enough to significantly destroy allergenic epitopes in eHWP.

The %CD63^+^ basophils/anti-FcεRI ratio was identified as an appropriate BAT parameter to differentiate reactivity to ATS between patients with WDEIA and control subjects. The discriminability estimated from the AUC of each ROC curve varied between gluten samples and HWP, with the best results for sHWP (AUC ROC: 0.925). Specificity (83%) and sensitivity (100%) of %CD63^+^ basophils/anti-FcεRI ratio using sHWP at optimal cut-off were very good. Another advantage is that ATS preparation was easy and fast and did not require complex extractions or enzymatic digestions, as in the case of gluten isolates (20, 34, 55). We found no correlations between BAT results (CD63^+^ basophils, %CD63^+^ basophils/anti-FcεRI ratio), diameter of wheals and erythema in SPT, sIgE, or disease severity. This is understandable regarding the levels of sIgE, because only clinical routine IgE determination for WDEIA was available. As mainly ATIs were identified in the ATS (G, G-ω5, and sHWP), the allergic reactions to these are found here and those did not appear to be related to sIgE against wheat flour, gluten, gliadin, ω5-gliadin, or LTP. Further insights into possible correlations between BAT and sIgE levels could be gained by measurements of sIgE against ATI types. One possible reason for the lack of correlation between BAT results and disease severity may be that basophil granulocytes are only one part of the whole allergic reaction that has many other influencing factors (e.g., mast cells) (56).

Three of the twelve patients (p5, p7, and p8) showed low basophil activations to G, G-ω5, sHWP, and eHWP in general. P8 showed low basophil activations to the ATS assessed here, but showed a high reactivity to ω5-gliadins in our previous study (20). Patients p5 and p7 had low basophil reactions in the present and in our previous study (20). The IgE positive control showed a basophil activation in both cases, but the basophil granulocytes did not react to the allergens tested in either case. The exact reasons remain unclear at this point in time, but warrant further investigations.

In our previous study, we investigated the basophil activity to isolated ω5-gliadins in the context of WDEIA with the same patients and controls as in the present study. The BAT parameter %CD63^+^ basophils was identified as the best parameter in this case to differentiate between patients and controls. Thereby, the ATS from isolated ω5-gliadins showed a test sensitivity of 100%, a specificity of 90%, and an AUC ROC of 0.975 at a concentration of 4 mg/ml (20). In comparison with G, G-ω5, and eHWP, the results for ω5-gliadins were better, but comparable with sHWP (sensitivity 100%, specificity 83%, and AUC ROC 0.925). As we identified high amounts of ATI in the ATS of sHWP, this underlines the result of the present study, that non-gluten proteins carrying yet unidentified allergenic epitopes appear to be relevant in WDEIA.

One acknowledged limitation of our study is the comparatively small number of patients with WDEIA and controls. The main reason is that the prevalence of WDEIA is very low overall and the participants were only recruited from the surrounding area of one specialized center. Our main intent was to identify the causative proteins in the ATS first, before we continue studies with more patients with WDEIA from several centers.

In conclusion, we found differences in allergenicity of gluten and HWP samples with varying molecular composition in individual patients with WDEIA using SPT and BAT. The %CD63^+^ basophils/anti-FcεRI ratio was the most promising parameter to distinguish patients from controls. The procedure to prepare ATS from sHWP is easy and can be performed even in routine clinical practice to help establish BAT as another option to complement the WDEIA diagnosis. Since the ATS made of G, G-ω5, and sHWP predominantly contained ATIs and only small concentrations of gluten proteins, more research is needed to clarify the role of non-gluten proteins in WDEIA and identify specific mechanisms of immune activation.

## Data Availability Statement

The original contributions presented in the study are included in the article/Supplementary Material, further inquiries can be directed to the corresponding author/s.

## Ethics Statement

The studies involving human participants were reviewed and approved by Ethics Committee, Technical University of Munich. The patients/participants provided their written informed consent to participate in this study.

## Authors Contributions

AG, JG, M-CN, BE, TB, KS, and KB: conceptualization. AG, JG, and M-CN: formal analysis, investigation, and methodology. AG, JG, and BE: data curation. BE, TB, KS, and KB: funding acquisition, resources, and supervision. AG: visualization and writing—original draft. JG, M-CN, BE, TB, KS, and KB: writing—review and editing. All authors read and approved the final manuscript.

## Funding

This work was supported by the authors' institutes, by research and development grants from the German Federal Ministry of Education and Research (BMBF), project ABROGATE (funding number: 01EA2106A), and the Deutsche Forschungsgemeinschaft (DFG; German Research Foundation) CRC 1371 P06; bilateral funding from Luxembourg National Research Fund (FNR) project C17/BM/11656090—DFG DACH-Lead-Agency BI696/12-1.

## Conflict of Interest

BE received methodological and technical support from Bühlmann Laboratories AG (Schönenbuch, Switzerland). The remaining authors declare that the research was conducted in the absence of any commercial or financial relationships that could be construed as a potential conflict of interest.

## Publisher's Note

All claims expressed in this article are solely those of the authors and do not necessarily represent those of their affiliated organizations, or those of the publisher, the editors and the reviewers. Any product that may be evaluated in this article, or claim that may be made by its manufacturer, is not guaranteed or endorsed by the publisher.
