# Supplementary material for: Basophil Activation to Gluten and Non-Gluten Proteins in Wheat-Dependent Exercise-Induced Anaphylaxis
Source: Front Allergy. 2022 Feb 25;3:822554. doi: 10.3389/falgy.2022.822554 (PMC8974719; doi:10.3389/falgy.2022.822554)
Supplement: Supplementary file 1 [file Data_Sheet_1.PDF]

SUPPORTING INFORMATION

Basophil activation to gluten and non-gluten proteins in wheat-dependent exercise-induced anaphylaxis

Angelika Miriam Gabler[1], Julia Gebhard[2], Marie-Christin Norwig[1], Bernadette Eberlein[2], Tilo Biedermann[2], Knut Brockow[2], Katharina Anne Scherf[1,3]

[1] Leibniz-Institute for Food Systems Biology at the Technical University of Munich, Freising, Germany

[2] Department of Dermatology and Allergy Biederstein, TUM School of Medicine, Technical University of Munich (TUM), Munich, Germany

[3] Department of Bioactive and Functional Food Chemistry, Institute of Applied Biosciences, Karlsruhe Institute of Technology (KIT), Karlsruhe, Germany

Table S1. Parameters used for data analysis for protein identification in MaxQuant. Three different searches against databases of the *Triticum aestivum* proteome (downloaded from the UniprotKB database, http://uniprot.org; version 2019/08) were carried out considering the enzymes used for digestion during sample preparation (pepsin, trypsin, chymotrypsin, thermolysin).

	**Search 1**	**Search 2**	**Search 3**
**Digestion mode**	specific[a]	specific[a]	unspecific[c]
**Maximum missed cleavages**	10	10	-
**Protein false discovery rate**	1.00	0.01	0.01
**Protein data base**	*Triticum aestivum*	*Triticum aestivum* app.[b]	*Triticum aestivum* app.[b]

[a] pepsin, trypsin, chymotrypsin, thermolysin (depending on the enzymes used for digestion, respectively), [b] appropriate FASTA files were generated on the basis of search 1 using the proteinGroups.txt files, [c] minimum/maximum peptide length: 4/50

Table S2. Skin prick test for WDEIA patients (p): gluten samples G and G-ω5 and hydrolyzed wheat proteins sHWP and eHWP. Histamine dihypochloride (10%) solution was used as positive control and isotonic sodium chloride solution as negative control (NaCl). The diameter of wheals (W) and erythema (E) were documented in mm. The outcome is classified as positive reaction (blue), when the diameter of the wheal caused by the tested substance is greater than or equal to the diameter of the wheal caused by the negative control with 3 mm in addition. The results are the average of two determinations (except patient 4, n = 1).

	**G[a]**		**G-ω5**		**sHWP**		**eHWP**		**Histamine[a]**		**NaCl[a]**	
**p**	**W**	**E**	**W**	**E**	**W**	**E**	**W**	**E**	**W**	**E**	**W**	**E**
**1**	6.5	14.0	0.5	2.0	4.0	10.0	1.0	2.0	6.0	8.0	0.0	0.0
**2**	9.0	15.0	5.5	8.5	7.5	12.5	16.5	20.0	6.0	9.0	0.0	2.0
**3**	6.0	17.0	6.0	9.5	9.5	16.5	11.0	17.5	7.0	10	0.0	0.0
**4**	7.0	10.0	5.0	6.0	4.0	4.0	7.0	8.0	6.0	8.0	0.0	0.0
**5**	4.0	14.0	3.0	5.0	6.0	10.0	4.0	5.0	6.0	8.0	0.0	2.0
**6**	6.0	14.0	4.0	6.0	6.0	11.0	9.5	17.5	6.0	13.0	0.0	0.0
**7**	5.5	11.0	1.0	3.0	2.5	5.5	1.5	2.0	6.0	11.0	0.0	0.0
**8**	11.5	31.0	5.0	13.5	9.5	29.0	6.5	20.5	6.0	19.0	0.0	2.0
**9**	3.5	10.0	4.0	13.5	6.5	10.0	4.5	12.0	6.0	21.0	0.0	0.0
**10**	4.4	21.0	3.0	3.5	4.5	23.0	2.0	2.5	4.0	15.0	0.0	0.0
**11**	6.5	21.0	5.0	10.0	5.5	14.5	8.0	23.5	6.0	21.0	0.0	2.0
**12**	3.0	7.0	3.0	3.0	4.0	18.0	2.5	3.0	5.0	18.0	0.0	1.0
**range**	3.5-11.5	7.0-31.0	0.5-6.0	2.0-13.5	2.5-9.5	4.0-29.0	1.0-16.5	2.0-23.5	4.0-7.0	8.0-19.0	0.0	0.0-2.0
**mean**	6.1	15.4	3.8	7.0	5.8	13.7	6.2	11.1	5.8	13.4	0.0	0.8
**median**	6.0	14.0	4.0	6.0	5.8	11.8	5.5	10.0	6.0	12.0	0.0	0.0

[a] The values in these columns were added for comparison and are already reported in Gabler *et al.* 2021 (DOI: 10.1002/clt2.12050).

Table S3. Area under the curve (AUC) of receiver operating characteristic curves from WDEIA patients and controls for G, G-ω5, sHWP and eHWP including sensitivity, specificity and optimal discrimination threshold (cut-off) for %CD63$^+$ basophils/anti-FcεRI ratio, when a basophil activation is classified as allergic response to an allergen test solution (ATS).

ATS	AUC	Cut-off %CD63$^+$ basophils/anti-FcεRI ratio	Sensitivity [%]	Specificity [%]
sHWP	0.925	0.197	100	83
G-ω5	0.783	0.035	92	60
G	0.775	0.030	92	70
eHWP	0.692	0.044	75	70

Table S4. Proteins identified in allergen test solution (ATS) of gluten G by UPLC-TripleTOF-MS after digestion with pepsin and trypsin. MaxQuant search parameters were: protein false discovery rate, 0.01; digestion mode: specific.

Name	UniProt Accession	SC [%]	Score	Intensity	No. of peptides	No. of unique peptides	Peptide sequences
CM16 ATI	P16159	41.3	313.9	32‘405‘000	8	8	ANIPQQCR, IETPGSPYL, ILVTPGYCNL, ITPLPSCR, MELPGCPR, SRPDQSGL, TTVHNTPY, VTPGYCNL
AAI-DCP	A0A3B6JR20	44.6	276.6	39‘579‘000	10	3	SGNVGESGLAEIPQQCR, ATIHNVR, IALPVPSQPVDPR, IDLPGCPR, LVAPGQCNL, PVPSQPVDPR, SGNVGESGL, SPMKPYL, YCCQEL, YCPAVEQL
Mono-meric-AAI	A4ZIZ6	43.0	85.4	14‘759‘000	6	5	EVLPGCR, QCVGSQVPEAVL, RDCCQQL, SVYQEL, TAASVPEVCK, VPIPNPSGDR
LMW-GS	I1XB60	20.1	64.3	1‘612‘300	6	2	PQIPQQSR, PQQQIPF, SHHQQQQPIQQQPQPF, SQQQPCSQQQQQPLSQQQQPPF, TTTSVPF, VHPSIL
CM1 ATI	P16850	20.8	60.4	5‘829‘600	5	2	DLPGCPR, KDLPGCPR, RSDPNSSVL, SDPNSSVL, YDASQHCR
Dimeric-AAI	L7P0X6	38.8	46.5	7‘528‘300	6	0	DVAAYPDA, EHGAQEGQAGTGAF, KLQCNGSQVPEAVL, PACRPL, PACRPLL, RDCCQQL

AAI: α-amylase inhibitor, ATI: α-amylase/trypsin inhibitor, DCP: domain-containing protein, No.: number, SC: protein sequence coverage.

Table S5. Proteins identified in allergen test solution (ATS) of gluten G by UPLC-TripleTOF-MS after digestion with pepsin and trypsin. MaxQuant search parameters were: protein false discovery rate, 0.01; digestion mode: unspecific.

Name	UniProt Accession	SC [%]	Score	Intensity	No. of peptides	No. of unique peptides	Peptide sequences
CM2 ATI	P16851	45.0	305.2	10‘356‘000	9	4	DLPGCPR, IVGSPVSTEPGNTPR, KDLPGCPR, TSDPNSGVL, VGIVGSPVSTEPGNTPR, VGIVGSPVSTEPGNTPRD, VLVTPGHCNVM, VSTEPGNTPR, YVAQQTCG
CM16 ATI	P16159	39.2	203.9	31‘890‘000	12	3	ANIPQQCR, IETPGSPYL, ITPLPS, ITPLPSCR, ITPLPSCRD, LITPLPS, LITPLPSCR, MELPGCPR, PGYCNL, SRPDQSGL, VQMDF, VTPGYCNL
CM3 ATI	P17314	44.6	128.5	33‘298‘000	10	9	DLPGCPR, IALPVPSQPVDPR, IDLPGCPR, LVAPGQCNL, MQWDF, SGNVGESGL, SGSCVPGVAF, TIHNVR, YCCQEL, YCPAVEQPL
CM1 ATI	P16850	54.2	91.9	10‘656‘000	10	5	DLPGCPR, ISISGSA, KDLPGCPR, MGLPINPLEGCRE, PINPLEG, PINPLEGCRE, SDPNSSVL, VLVTPGHCNVM, VSTEPGNTPR, YVAQQTCG
0.53 ATI	P01084	49.2	60.9	27‘770‘000	8	0	EHGVSEGQAGTGAF, FQVPAL, FQVPALPGCR, FQVPALPGCRPLL, KLQCNGSQVPEAVL, LPIVVD, MCYPGQA, RDCCQQL
0.28 ATI	P01083	52.9	47.1	10‘759‘000	8	7	ASVPEVCK, EVLPGCR, QCVGSQVPEAVL, RDCCQQL, SVYQEL, SWCDPATG, VPIPNPSGDR, WAAYPD
α-gliadin	Q1WA40	20.1	41.1	1‘303‘300	6	4	LQIPEQSR, QPQSFP, QQQLIPCRD, VRVPVPQLQPKNPSQQQPQE, VRVPVPQLQPKNPSQQQPQEQVPLVQ, VSFQQPQQQYPSSQ

ATI: α-amylase/trypsin inhibitor, No.: number, SC: protein sequence coverage.

Table S6. Proteins identified in allergen test solution (ATS) of gluten G-ω5 by UPLC-TripleTOF-MS after digestion with pepsin and trypsin. MaxQuant search parameters were: protein false discovery rate, 0.01; digestion mode: specific.

**Name**	**UniProt Accession**	**SC [%]**	**Score**	**Intensity**	**No. of peptides**	**No. of unique peptides**	**Peptide sequences**
CM16 ATI	P16159	42.7	323.3	16‘076‘000	9	9	ANIPQQCR, IETPGSPYL, ILVTPGYCNL, ITPLPSCR, MELPGCPR, SRPDQSGL, TTVHNTPY, TTVHNTPYCL, VTPGYCNL
CM3 ATI	P17314	61.7	323.3	26‘790‘000	9	2	AEISQQCR, ATIHNVR, IALPVPSQPVDPR, IDLPGCPR, LVAPGQCNL, QQTCGTFTPGSKL, SGNVGESGL, YCCQEL, YCPAVEQPL
ω-gliadin[a]	C0KEH9[a]	24.2	120.1	706‘200	3	3	PLQPQQPFPQPQQPIPQQPQQSF, PQQPQQIIPQQTQQPFPLQPQQPFPQQPQRPF, QELQSPQQPVPK
CM2 ATI	P16851	21.4	81.7	3‘700‘500	4	1	CEAVRY, KDLPGCPR, TSDPNSGVL, YDASQHCR
α-gliadin	Q9M4M6	6.0	47.4	2‘190‘600	2	2	FQIPEQSR, VRVPVPQL
0.19 ATI	Q5MD68	27.9	45.3	5‘310‘600	5	4	DVAAYPDA, KLQCNGSQVPEAVL, PACRPL, PACRPLL, RDCCQQL
Proteinkinase-DCP	A0A3B6AVL7	7.6	45.0	969‘010	2	2	LLRLPF, MGGCHAKPRTRDADGASPPPPAPATPPPPSSTAPATPASK

[a] contains the WDEIA epitope QQPGQ, as reported by Matsuo *et al*. 2005, doi: 10.4049/jimmunol.175.12.8116, ATI: α-amylase/trypsin inhibitor, DCP: domain-containing protein, No.: number, SC: protein sequence coverage.

Table S7. Proteins identified in allergen test solution (ATS) of gluten G-ω5 by UPLC-TripleTOF-MS after digestion with pepsin and trypsin. MaxQuant search parameters were: protein false discovery rate, 0.01; digestion mode: unspecific.

Name	UniProt Accession	SC [%]	Score	Intensity	No. of peptides	No. of unique peptides	Peptide sequences
CM3 ATI	P17314	59.5	237.1	47‘819‘000	13	13	ASIYSPGKPYL,IALPVPSQPVDPR, IDLPGCPR, IDLPGCPRE, IYSPGKPYL, LVAPGQCNL, MQWDF, QQTCGTFTPGSKL, SGNVGESGL, SGSCVPGVAF, TIHNVR, YCCQEL, YCPAVEQPL
CM16 ATI	P16159	46.2	212.2	30‘793‘000	14	5	ANIPQQCR, DCTPWM, IETPGSPYL, ILVTPGYCNL, ITPLPSCR, ITPLPSCRD, LITPLPSCR, LITPLPSCRD, MELPGCPR, MELPGCPRE, PGYCNL, TTVHNTPYC, VQMDF, VTPGYCNL
CM2 ATI	P16851	36.7	98.7	15‘676‘000	6	3	IVGSPVSTEPGNTPR, KDLPGCPR, LVTPGHCNVM, TSDPNSGVL, VGIVGSPVSTEPGNTPR, VSTEPGNTPR
ω-gliadin[a]	C0KEH9[a]	23.8	97.4	4‘863‘800	7	7	ELQSPQQPVPK, IIPQQPQQPFPLQ, IISQQPFPLQPQQPF, IISQRPQQPFPLQ, IISQRPQQPFPLQPQQPF, QELQSPQQPVPK, QIISQRPQQPFPLQPQQ
α-gliadin	Q9M4M3	25.5	53.5	5‘127‘900	9	2	FQIPEQSR, IAPFGIF, LCCQQL, PLVQQQQFLG, QQQLIPCRD, RCQAIHNVVHA, VRVPVPQL, VRVPVPQLQPQNPSQQQPQE, VRVPVPQLQPQNPSQQQPQEQVPLVQ

[a] contains the WDEIA epitope QQPGQ, as reported by Matsuo *et al*. 2005, doi: 10.4049/jimmunol.175.12.8116, ATI: α-amylase/trypsin inhibitor, No.: number, SC: protein sequence coverage.

Table S8. Proteins identified in allergen test solution (ATS) of hydrolyzed wheat protein sHWP by UPLC-TripleTOF-MS after digestion with pepsin and trypsin. MaxQuant search parameters were: protein false discovery rate, 0.01; digestion mode: specific.

Name	UniProt Accession	SC [%]	Score	Intensity	No. of peptides	No. of unique peptides	Peptide sequences
CM16 ATI	P16159	25.2	323.3	5‘315‘600	7	3	ANIPQQCR, ILVTPGYCNL, ITPLPSCR, ITPLPSCRD, LITPLPSCRD, MELPGCPR, VTPGYCNL
CM3 ATI	P17314	50.4	323.3	8‘624‘100	8	1	IALPVPSQPVDPR, IDLPGCPR, LPVPSQPVDPR, LVAPGQCNL, QQTCGTFTPGS
GSP-1 protein	Q84QI5	20.7	231.1	1‘772‘600	5	3	CNIPITSG, DMPLSW, EAKTVQTAK, TSIQGDLSG, TSIQGDLSGFK
dimeric-AAI	I6PZ03	47.5	153.6	3‘920‘900	8	0	DVAAYPDA, FQVPAL, KLQCNGSQVPEAVL, LPIVVDASGDGAY, MCYPGQA, PACRPLL, QCNGSQVPEAVL, RDCCQQLAHISE
γ-gliadin	Q6EEW7	11.7	109.8	521‘220	3	1	FPQLQQPQQP, PQPHQP, VQGQGIIQPQQPAQL
LMW-GS	Q5MFN4	16.7	79.8	2‘034‘100	6	1	LSHHQQQQPIQQQPQPF, LVLPQQQIPF, PQQPSF, PQQQIPF, PQQQIPFVHPSIL, RTTTSVPFGVGTGVGS
AAI-DCP	A0A3B5XTA4	8.5	74.7	4‘954‘200	2	2	IAVIGT, QQQQQL
Lipoxygenase	A0A3B6LFW8	0.8	67.1	584‘090	1	1	LLGDVRIF
0.28 ATI	P01083	26.5	59.1	504‘940	3	3	QCVGSQVPEAVL, VATTMAVEYGARSHNSGP, VPIPNPSGDR
UP (BED-type-DCP[a])	A0A3B6CD58 (A0A446MQD1[a])	2.3	42.9	812‘470	3	3	CAHITTVLLPMEEEENNL, SIVTGSA, YARIDGLQCKKIL

[a] 90% similarity, ATI: α-amylase/trypsin inhibitor, DCP: domain-containing protein, GSP: grain softness protein, LMW-GS: low-molecular-weight glutenin-subunit No.: number, SC: protein sequence coverage, UP: uncharacterized protein.

Table S9. Proteins identified in allergen test solution (ATS) of hydrolyzed wheat protein sHWP by UPLC-TripleTOF-MS after digestion with pepsin and trypsin. MaxQuant search parameters were: protein false discovery rate, 0.01; digestion mode: unspecific.

Name	UniProt Accession	SC [%]	Score	Intensity	No. of peptides	No. of unique peptides	Peptide sequences
CM3 ATI	P17314	54.8	323.3	12‘396‘000	11	2	ASIYSPGKPYL, IALPVPSQPVD, ASIYSPGKPYL, IALPVPSQPVD, LVAPGQCNL, MQWDF, QQTCGTFTPGSKL, SGNVGESGL, SGSCVPGVA, YCCQELYCPAVEQPL
CM16 ATI	P16159	40.6	225.5	8‘267‘000	11	3	ANIPQQCR, EESQWS, IETPGSPYL, ILVTPGYCNL, ITPLPSCRD, LITPLPSCR, LITPLPSCRD, MELPGCPR, NEDCTPW, PGYCNL, VTPGYCNL
CM1 ATI	P16850	40.0	188.5	3‘771‘400	7	4	ISISGSA, KDLPGCPR, LVTPGHCNVM, PINPLEG, PINPLEGCRE, SVLKDLPGCPREP, YVAQQTCG
1B2-protein	Q5BLQ7	20.4	77.1	2‘912‘500	5	4	CNIPITSG, DMPLSW, QCCQQL, TSIQGDL, TSIQGDLSG
CM2 ATI	P16851	41.7	67.9	2‘429‘600	5	2	IVGSPVSTEPGNTPR, KDLPGCPR, LVTPGHCNVM, TSDPNSGVL, YVAQQTCG
0.19 ATI	P01085	38.7	60.9	10‘305‘000	8	1	ASGDGAY, DVAAYPDA, FQVPAL, KLQCNGSQVPEAVL, LPIVVD, LPIVVDASGDGAY, MCYPGQA, QCNGSQVPEAVL
UP (F-box DCP[a])	A0A077RP85 (A0A3B6RAX4[a])	5.7	52.0	800‘000	1	1	TLRSLSFEDSDIPNILNTCNKL
gliadin-like seed protein	D2KFG9	22.4	45.5	1‘354‘000	7	1	DVHVPPYCY, IAVIGT, QCCQQL, QQQQQL, TTISPSSD, WVIQTI, WVIQTIPAMC

[a] 50% similarity, ATI: α-amylase/trypsin inhibitor, DCP: domain-containing protein, No.: number, SC: protein sequence coverage, UP: uncharacterized protein.

Table S10. Proteins identified in allergen test solution (ATS) of hydrolyzed wheat protein eHWP by UPLC-TripleTOF-MS after digestion with pepsin and trypsin. MaxQuant search parameters were: protein false discovery rate, 0.01; digestion mode: specific.

Name	UniProt Accession	SC [%]	Score	Intensity	No. of peptides	No. of unique peptides	Peptide sequences
LMW-GS	Q8W3X2	43.8	282.4	12‘804‘000	12	0	GVGTGVGGY, LQPHQIAQL, LQQQIPF, LQQQIPFVHPSIL, PPFSQQQQPVL, PQIPQQSR, PQQPPFSQQQLPPFSQQL, PQQPPFSQQQQPVL, PQQPPFSQQQQQPIL, PQQPSF, SQQQLPPFSQQLPPFSQQQQPVL, VHPSIL
α-gliadin	Q306G0	13.5	186.6	11‘919‘000	5	0	GIFGTN, LQIPEQSR, QLLQQL,QPQNPSQQQPQEQVPL, VQQQQF
γ-gliadin	Q94G97	15.2	113.0	26‘234‘000	5	0	AQIPQQL, EGIRSL, GIIQPQQPAQL, LQQCNPVSL, LQQQMNPCK
α/β-gliadin	P18573	14.6	92.1	7‘882‘200	5	0	GIFGTN, QLLQQL, QPQNPSQQQPQEQVPL, VQQQQF, WQIPEQSR
UP (Neprosin-DCP[a])	A0A3B6DAX2 (T1N2S6[a])	2.1	59.9	4‘615‘000	1	1	ICCAGQL
CM3 ATI	P17314	21.1	43.8	6‘230‘600	3	3	ATIHNVR, IALPVPSQPVDPR, IDLPGCPR
Adenosine-triphosphatase	A0A3B6RR54	8.4	41.9	11‘004‘000	2	2	AAAFVRAPLGASDKERHALVIRRDFDSGY, GMARDILPPELR

[a] 50% similarity, ATI: α-amylase/trypsin inhibitor, DCP: domain-containing protein, No.: number, SC: protein sequence coverage, UP: uncharacterized protein.

Table S11. Proteins identified in allergen test solution (ATS) of hydrolyzed wheat protein eHWP by UPLC-TripleTOF-MS after digestion with pepsin and trypsin. MaxQuant search parameters were: protein false discovery rate, 0.01; digestion mode: unspecific.

Name	UniProt Accession	SC [%]	Score	Intensity	No. of peptides	No. of unique peptides	Peptide sequences
α-gliadin	Q41530	35.7	323.3	26‘121‘000	16	0	FQIPEQSR, IAPFGIF, ILQQQLIPCRD, LCCQQL, LGQGSFR, LQPFPQPQLPY, LQPQNPSQQQPQEQVP, LQPQNPSQQQPQEQVPLVQQQ, LQPQQP, LVQQQQF, QILQQQLIPCRD, QPQSFP, QQQLIPCRD, VQQQQF, VVLQQH, VYIPPYCT
LMW-GS	B2Y2R5	36.7	323.3	25‘546‘000	21	0	CQQLPQIPQQSR, GLERPSQQQPLPPQQT, GVGTGVGGY, ILPQQPPFSQQQQ, LAQGTF, LERPSQQQP, LERPSQQQPLPPQQT, LPPFSQQQQPVL, LQPHQIAQL, LQQQIPF, LQQQIPFVHPSIL, LQQQLPP, PQIPQQSR, PQQPPFSQQQQPVL, PQQPPFSQQQQQPIL, PQQPSF, QQPILPQQPPF, TLPTMCN, VGTGVGGY, VHPSIL, VLPQQPPFSQQ
γ-gliadin	B6UKN7	27.4	212.7	7‘388‘700	17	0	AAIHSVVHS, AIHSVVH, AIHSVVHS, AQIPQQL, AQIPQQLQC, GIIQPQQPAQL, IIQPQQPAQL, LAQIPQQL, LQPQQP, LQQCNPVSL, LQQQMNPCK, PQQLQC, PQQPAQL, TIPQPHQT, VQWPQQQ, VQWPQQQPFPQPQQPF,VYVPPECS
γ-gliadin	Q9FS76	25.5	183.8	55‘919‘000	19	0	AAIHSVVHS, AIHSVVH, AIHSVVHS, AQIPQQL, AQIPQQLQC, GIIQPQQPAQL, IIQPQQPAQL, LAQIPQQL, LLQQCKPAS, LQPQQP, NIQVDPSGQ, NIQVDPSGQVQW, PFIQPSL, PQQLQC, PQQPAQL, QTLPTMCN, TLPTMCN
α-gliadin	Q1WA40	26.9	102.3	11‘653‘000	15	0	IAPFGIF, ILQQQLIPCRD, LCCQQL, LQIPEQS, LQIPEQSR, LQPQNPSQQQPQEQVP, LQPQNPSQQQPQEQVPLVQQQ, LQPQQP, LVQQQQF, QILQQQLIPCRD, QPQSFP, QQQLIPCRD, VQQQQF, VVLQQH,VYIPPHCSTT
CM1 ATI	P16850	20.8	60.1	1‘750‘500	2	2	KDLPGCPR, VGIVGSPVSTEPGNTPR

α/β-gliadin	D2T2K3	37	57.2	9‘646‘600	16	5	FVQPQQL, FVQPQQLPQF, IAPFGIF, ILQQQLIPCRD, LCCQQL, LQPQNPSQQQPQEQVP, LQPQNPSQQQPQEQVPLVQQQ, LVQQQQF, QILQQQLIPCRD, QQQLIPCRD, RCQAIHNVIHA, VQQQQF, VVLQQH, VYIPPHCSTT
α-gliadin	A0A0E3Z5 U5	30.3	54.4	11‘622‘000	15	0	IAPFGIF, ILQQQLIPCRD, LCCQQL, LGQGSFR, LQPFPQPQLPY, LQPQNPSQQQPQEQVP, LQPQNPSQQQPQEQVPLVQQQ, LVQQQQF, QILQQQLIPCRD, QQQLIPCRD, VQQQQF, VVLQQH, VYIPPYCT, WQIPEQS, WQIPEQSR

ATI: α-amylase/trypsin inhibitor, LMW-GS: low-molecular-weight glutenin subunits.

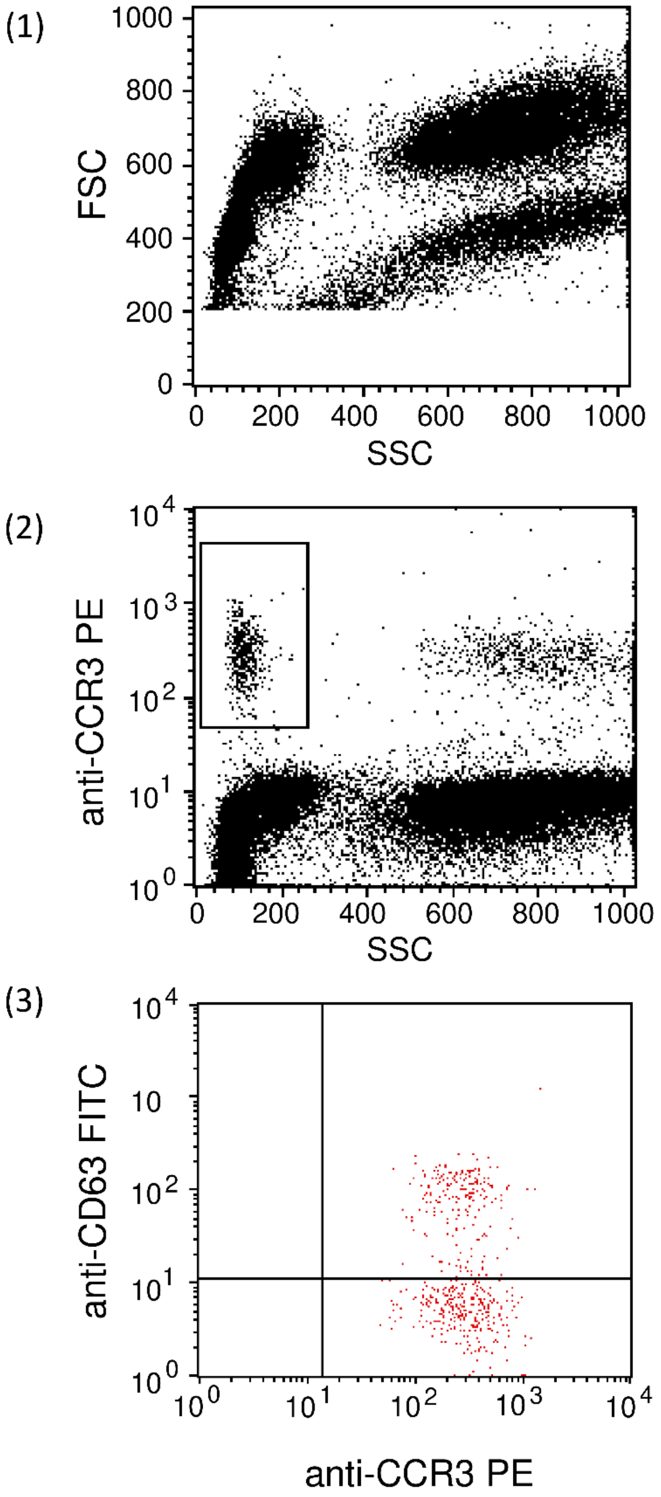

Figure S1. Basophil activation test measurement from patient 1 to the allergenic test solution of representative gluten (G). Flow cytometry was performed using a FACSCalibur system (Becton-Dickinson Immunocytometry System, Heidelberg, Germany) with a 488 nm, 15mW and a 635nm, 10mW argon laser. For basophil identification, the front (FSC) and side scatter (SSC) were used, as well as the identification marker CCR3, labeled with anti-CCR3-phycoerythrin (PE) mAb. The marked space in (2) shows the identified basophils. CD63 was used as basophil activation marker, which was labeled with anti-CD63-fluorescein-isothiocyanate (FITC) mAb. BD CellQuest (Becton-Dickinson Immunocytometry System) was used to analyze the data. At least 450 basophils were counted per measurement.

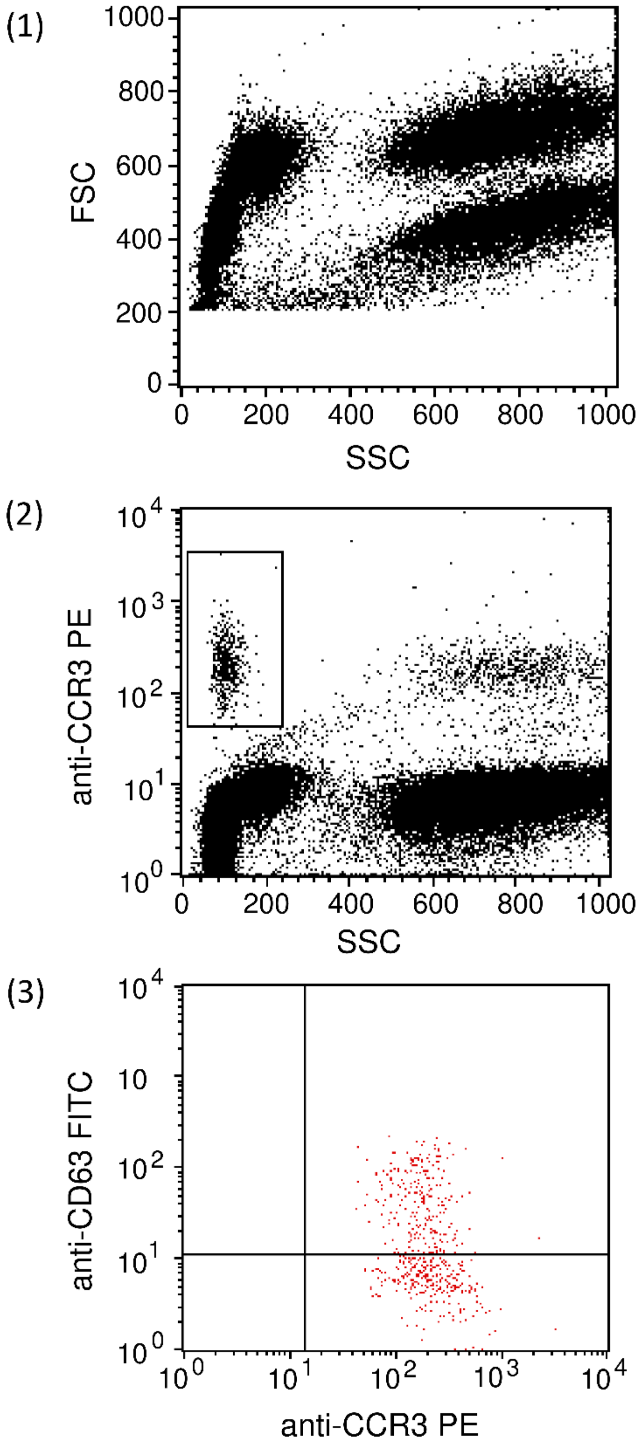

Figure S2. Basophil activation test measurement from patient 1 to the allergenic test solution of gluten with reduced ω5-gliadin content (G-ω5). Flow cytometry was performed using a FACSCalibur system (Becton-Dickinson Immunocytometry System, Heidelberg, Germany) with a 488 nm, 15mW and a 635nm, 10mW argon laser. For basophil identification, the front (FSC) and side scatter (SSC) were used, as well as the identification marker CCR3, labeled with anti-CCR3-phycoerythrin (PE) mAb. The marked space in (2) shows the identified basophils. CD63 was used as basophil activation marker, which was labeled with anti-CD63-fluorescein-isothiocyanate (FITC) mAb. BD CellQuest (Becton-Dickinson Immunocytometry System) was used to analyze the data. At least 450 basophils were counted per measurement.

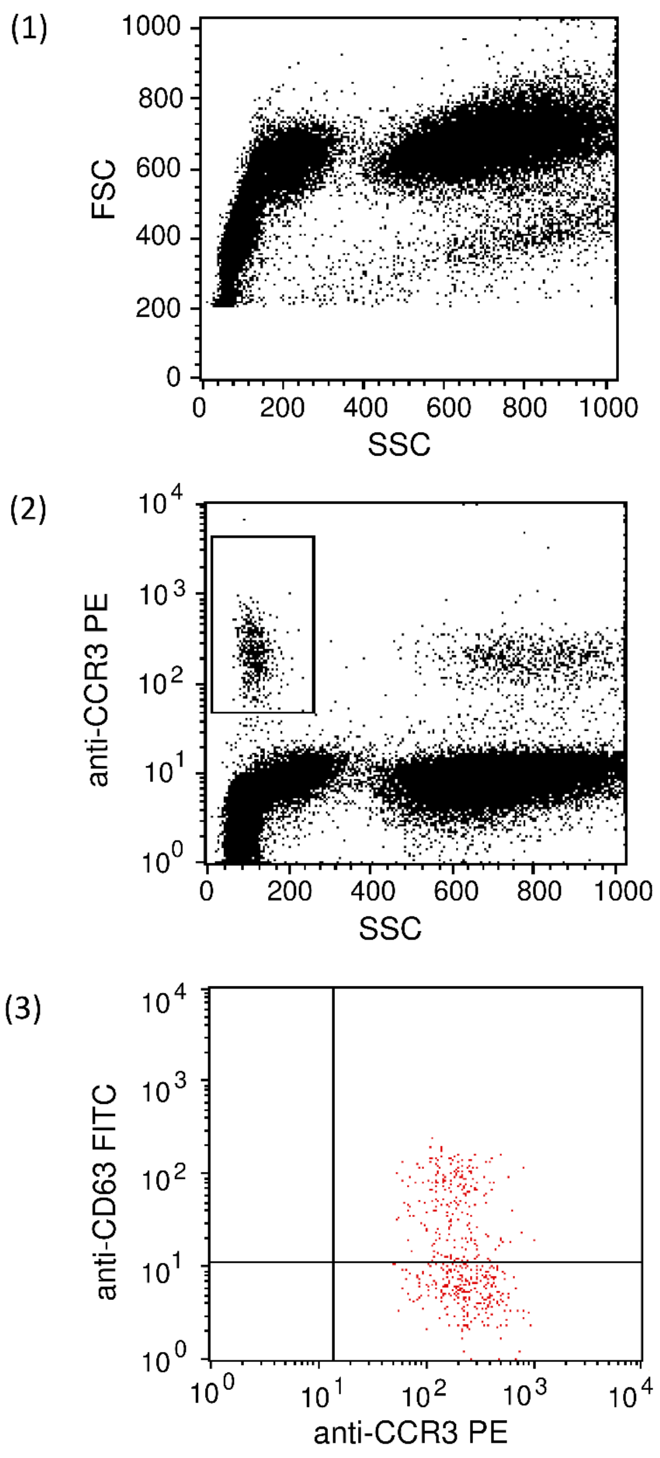

Figure S3. Basophil activation test measurement from patient 1 to the allergenic test solution of slightly hydrolyzed wheat protein (sHWP). Flow cytometry was performed using a FACSCalibur system (Becton-Dickinson Immunocytometry System, Heidelberg, Germany) with a 488 nm, 15mW and a 635nm, 10mW argon laser. For basophil identification, the front (FSC) and side scatter (SSC) were used, as well as the identification marker CCR3, labeled with anti-CCR3-phycoerythrin (PE) mAb. The marked space in (2) shows the identified basophils. CD63 was used as basophil activation marker, which was labeled with anti-CD63-fluorescein-isothiocyanate (FITC) mAb. BD CellQuest (Becton-Dickinson Immunocytometry System) was used to analyze the data. At least 450 basophils were counted per measurement.

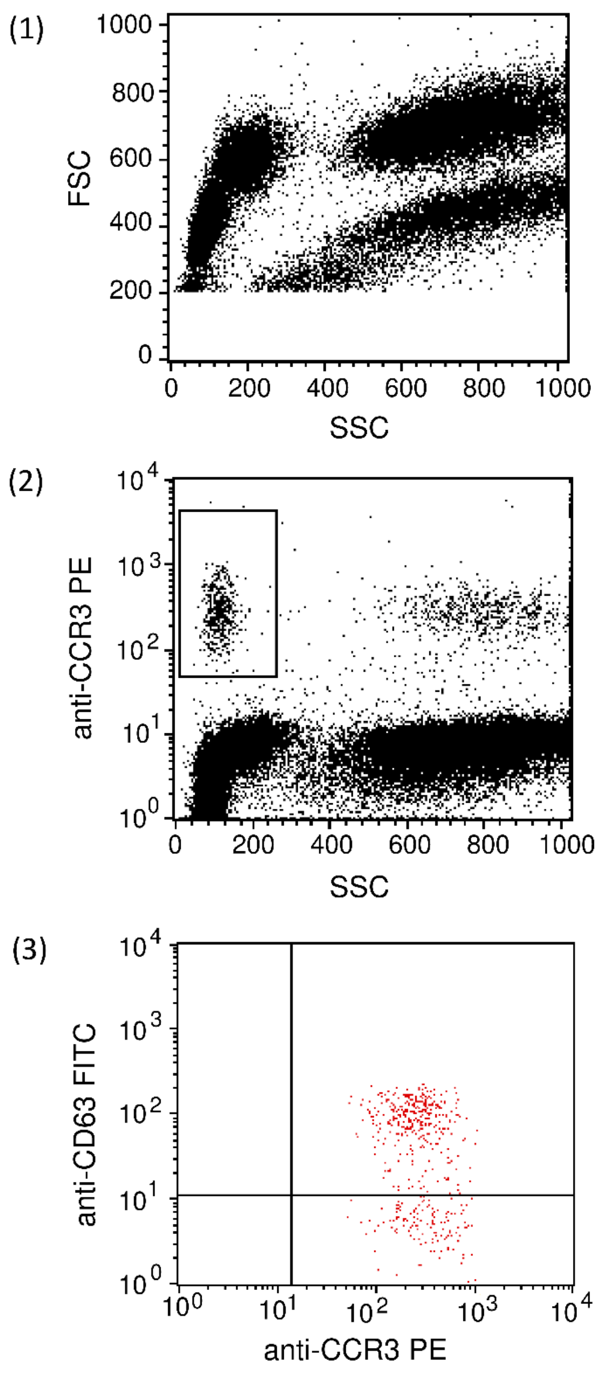

Figure S4. Basophil activation test measurement from patient 1 to the allergenic test solution of extensively hydrolyzed wheat protein (eHWP). Flow cytometry was performed using a FACSCalibur system (Becton-Dickinson Immunocytometry System, Heidelberg, Germany) with a 488 nm, 15mW and a 635nm, 10mW argon laser. For basophil identification, the front (FSC) and side scatter (SSC) were used, as well as the identification marker CCR3, labeled with anti-CCR3-phycoerythrin (PE) mAb. The marked space in (2) shows the identified basophils. CD63 was used as basophil activation marker, which was labeled with anti-CD63-fluorescein-isothiocyanate (FITC) mAb. BD CellQuest (Becton-Dickinson Immunocytometry System) was used to analyze the data. At least 450 basophils were counted per measurement.

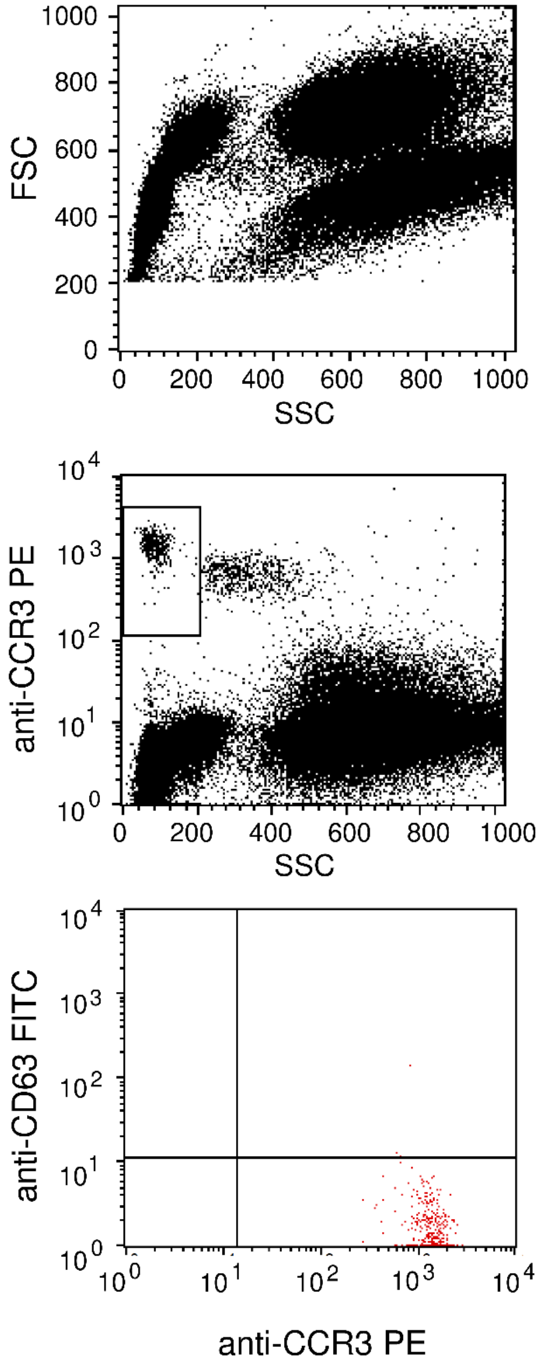

Figure S5. Basophil activation test measurement from control 1 to the allergenic test solution of representative gluten (G). Flow cytometry was performed using a FACSCalibur system (Becton-Dickinson Immunocytometry System, Heidelberg, Germany) with a 488 nm, 15mW and a 635nm, 10mW argon laser. For basophil identification, the front (FSC) and side scatter (SSC) were used, as well as the identification marker CCR3, labeled with anti-CCR3-phycoerythrin (PE) mAb. The marked space in (2) shows the identified basophils. CD63 was used as basophil activation marker, which was labeled with anti-CD63-fluorescein-isothiocyanate (FITC) mAb. BD CellQuest (Becton-Dickinson Immunocytometry System) was used to analyze the data. At least 450 basophils were counted per measurement.

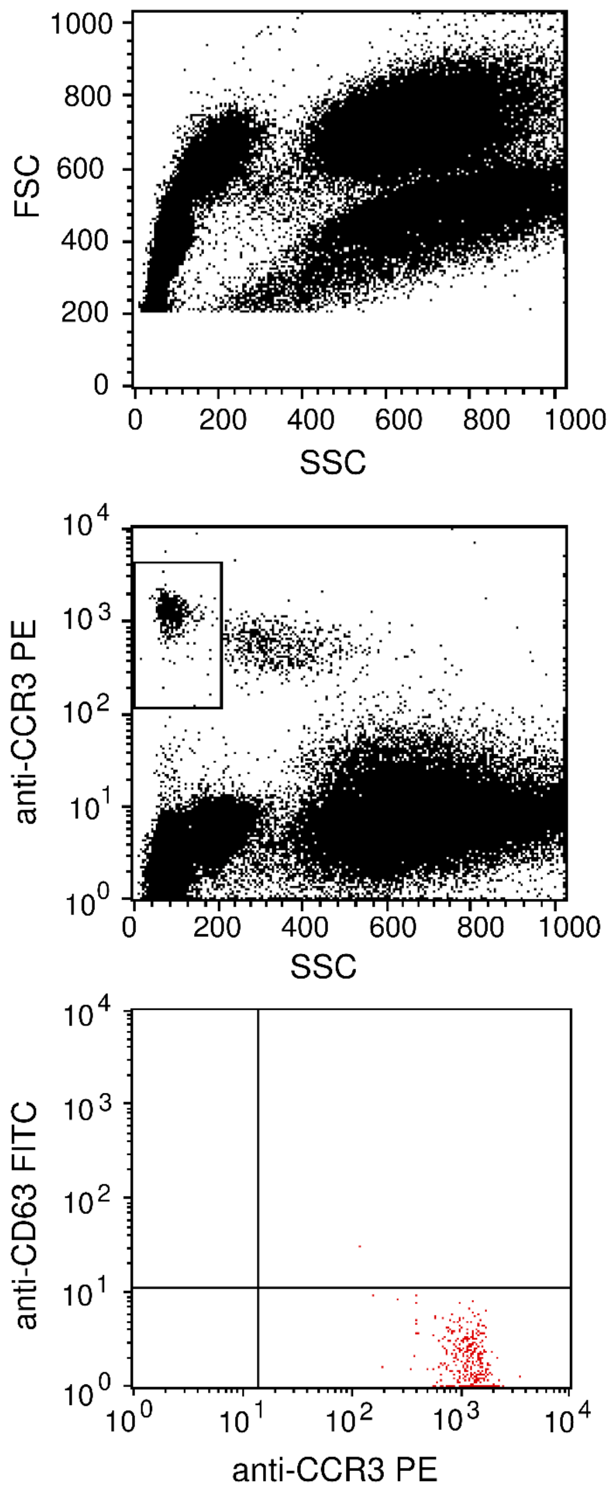

Figure S6. Basophil activation test measurement from control 1 to the allergenic test solution of gluten with reduced ω5-gliadin content (G-ω5). Flow cytometry was performed using a FACSCalibur system (Becton-Dickinson Immunocytometry System, Heidelberg, Germany) with a 488 nm, 15mW and a 635nm, 10mW argon laser. For basophil identification, the front (FSC) and side scatter (SSC) were used, as well as the identification marker CCR3, labeled with anti-CCR3-phycoerythrin (PE) mAb. The marked space in (2) shows the identified basophils. CD63 was used as basophil activation marker, which was labeled with anti-CD63-fluorescein-isothiocyanate (FITC) mAb. BD CellQuest (Becton-Dickinson Immunocytometry System) was used to analyze the data. At least 450 basophils were counted per measurement.

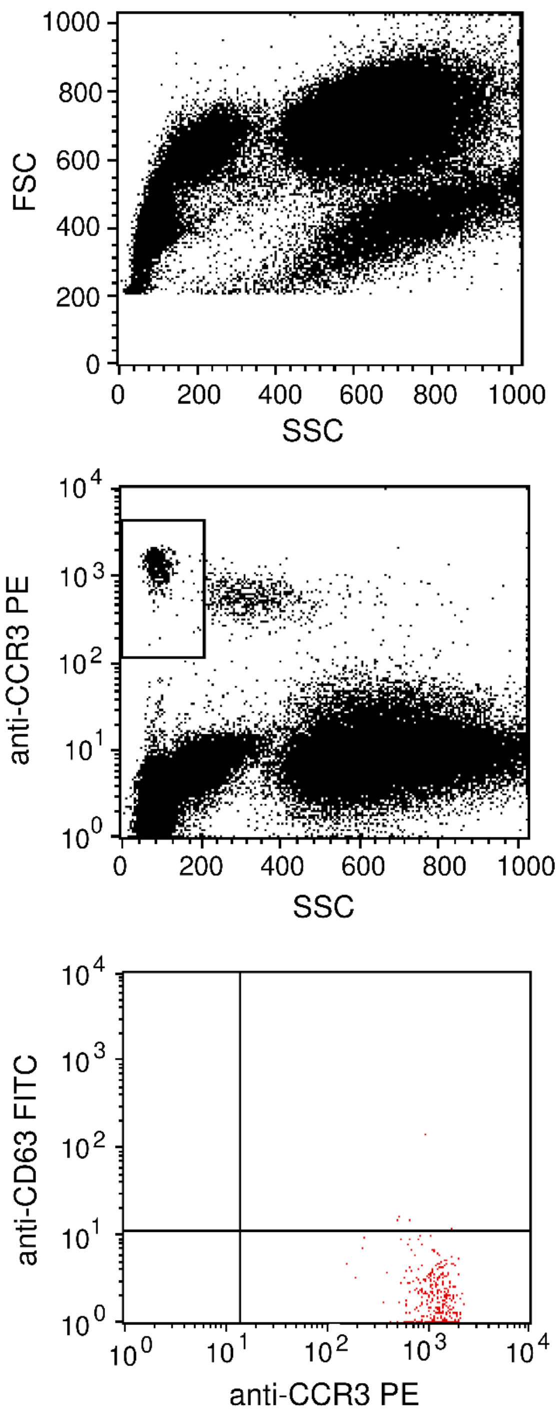

Figure S7. Basophil activation test measurement from control 1 to the allergenic test solution of slightly hydrolyzed wheat protein (sHWP). Flow cytometry was performed using a FACSCalibur system (Becton-Dickinson Immunocytometry System, Heidelberg, Germany) with a 488 nm, 15mW and a 635nm, 10mW argon laser. For basophil identification, the front (FSC) and side scatter (SSC) were used, as well as the identification marker CCR3, labeled with anti-CCR3-phycoerythrin (PE) mAb. The marked space in (2) shows the identified basophils. CD63 was used as basophil activation marker, which was labeled with anti-CD63-fluorescein-isothiocyanate (FITC) mAb. BD CellQuest (Becton-Dickinson Immunocytometry System) was used to analyze the data. At least 450 basophils were counted per measurement.

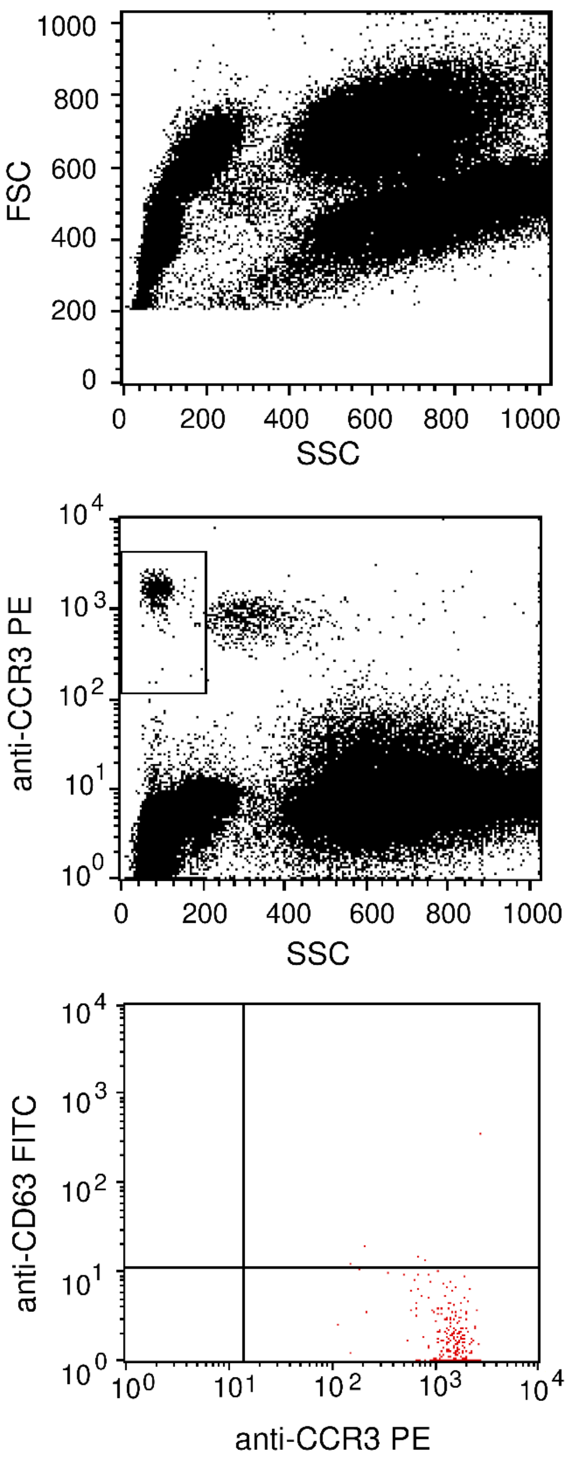

Figure S8. Basophil activation test measurement from control 1 to the allergenic test solution of extensively hydrolyzed wheat protein (eHWP). Flow cytometry was performed using a FACSCalibur system (Becton-Dickinson Immunocytometry System, Heidelberg, Germany) with a 488 nm, 15mW and a 635nm, 10mW argon laser. For basophil identification, the front (FSC) and side scatter (SSC) were used, as well as the identification marker CCR3, labeled with anti-CCR3-phycoerythrin (PE) mAb. The marked space in (2) shows the identified basophils. CD63 was used as basophil activation marker, which was labeled with anti-CD63-fluorescein-isothiocyanate (FITC) mAb. BD CellQuest (Becton-Dickinson Immunocytometry System) was used to analyze the data. At least 450 basophils were counted per measurement.

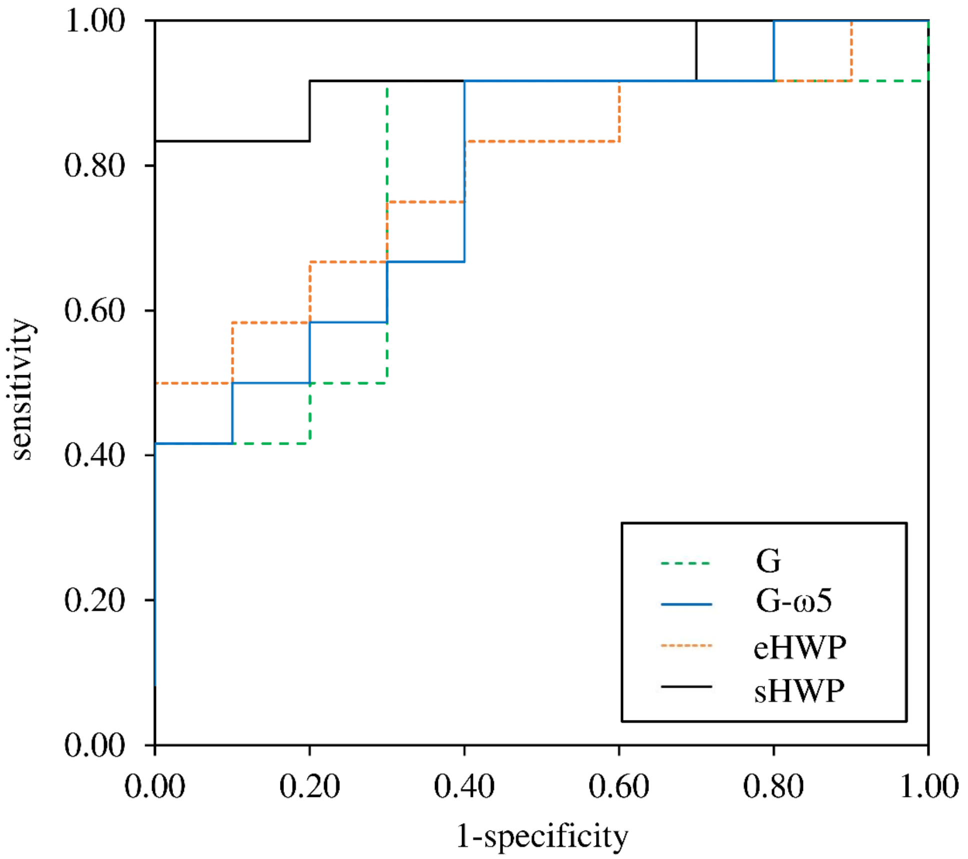

Figure S9. Receiver operating characteristic curves for G, G-ω5, sHWP and eHWP using the %CD63^{+} basophils/anti-FcεRI ratio for patients and controls.

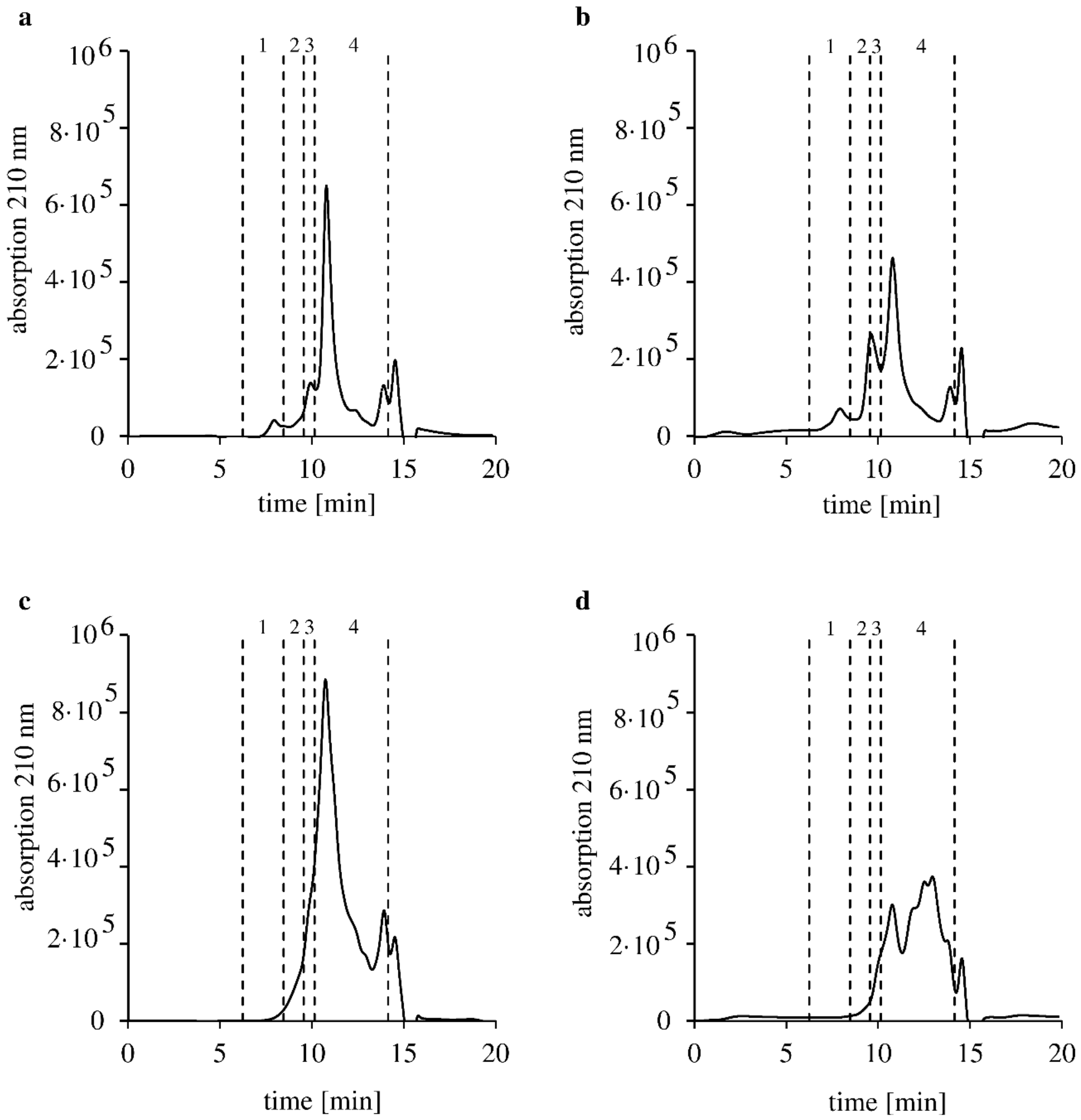

Figure S10. Gel permeation high-performance liquid chromatography: Chromatograms of the allergen test solutions G (a), G-ω5 (b), eHWP (c) and sHWP (d) using system I. System I is subdivided into the following ranges of relative molecular masses (M_r): 200-66 kDa (1), 66-29 kDa (2), 29-14 kDa (3), <14 kDa (4).

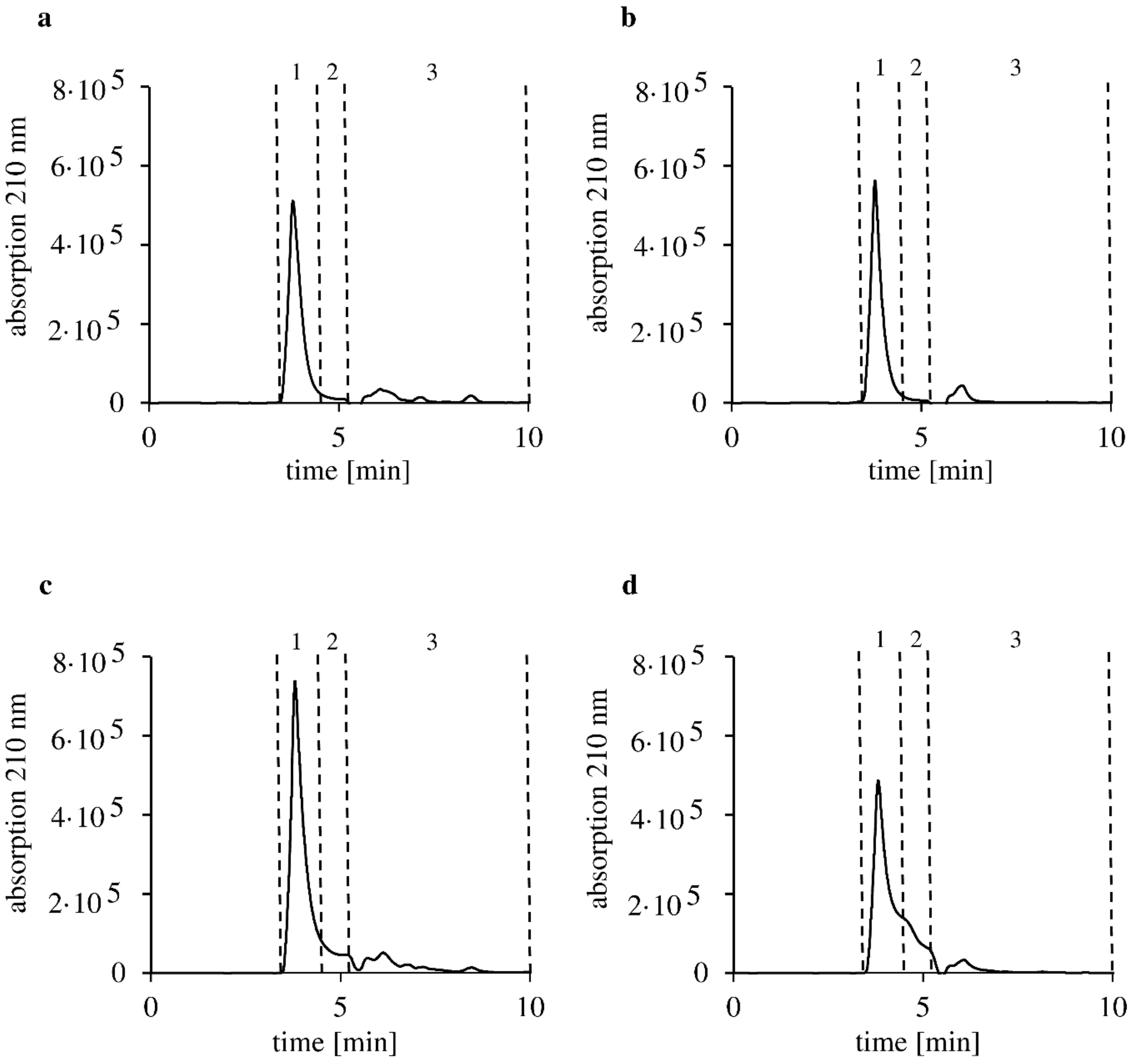

Figure S11. Gel permeation high-performance liquid chromatography: Chromatograms of the allergen test solutions G (a), G-ω5 (b), eHWP (c) and sHWP (d) using system II. System II is subdivided into the following ranges of relative molecular masses (M_r): ≥ 14 kDa (1), 14-2 kDa (2), <2 kDa (3).

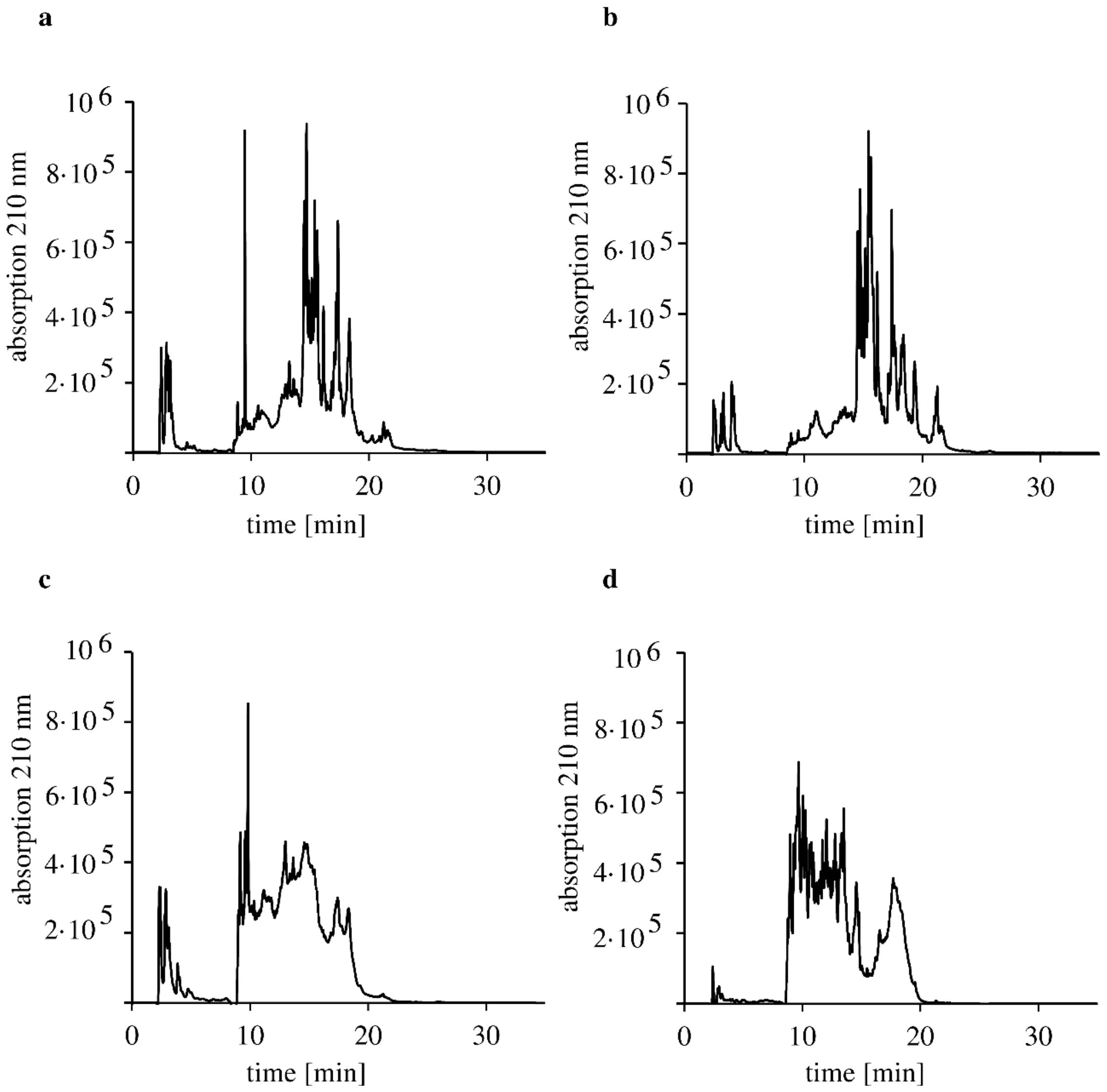

Figure S12. Reversed-phase high-performance liquid chromatography: Chromatograms of the allergen test solutions G (a), G-ω5 (b), eHWP (c) and sHWP (d).

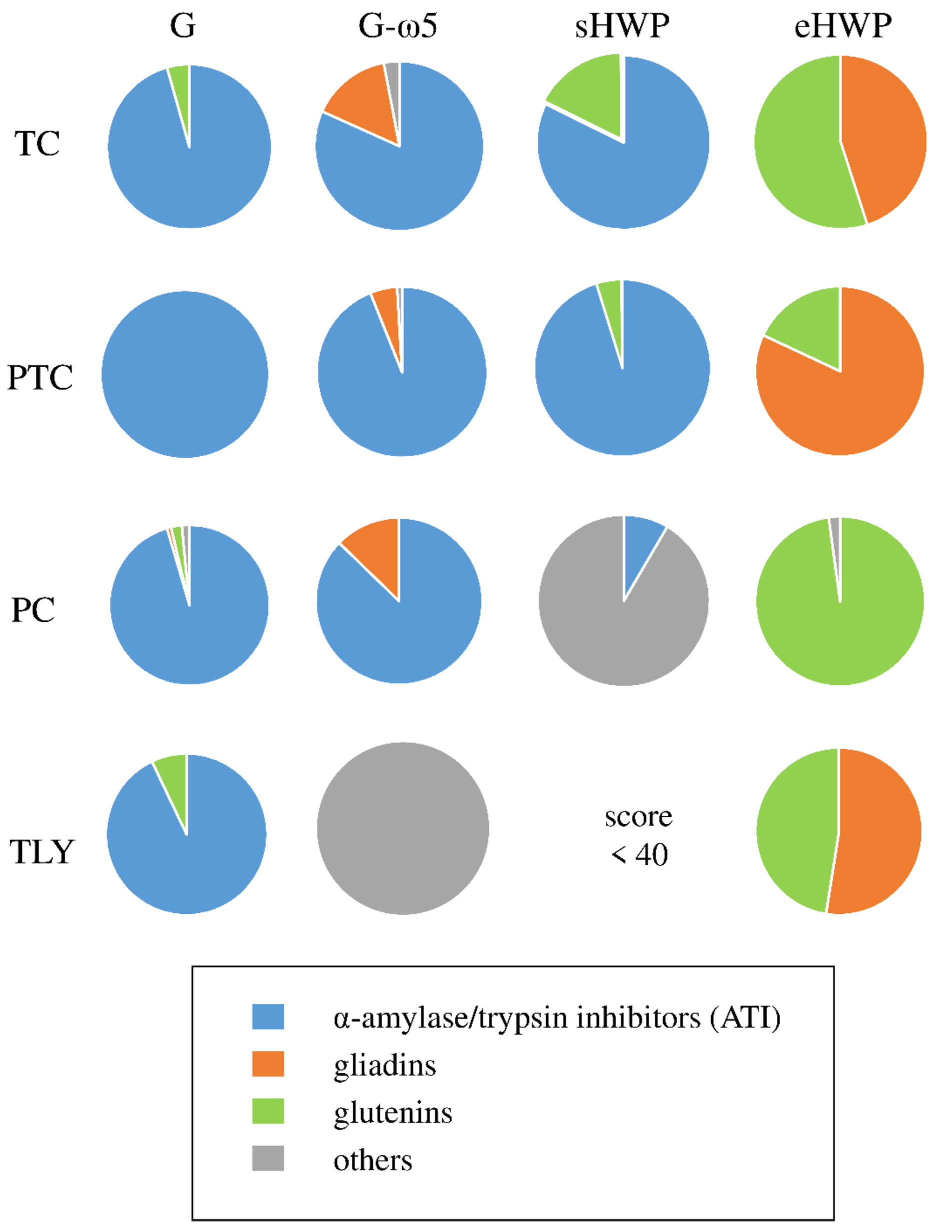

Figure S13. Percentages of the protein groups (protein score > 40) including α-amylase/trypsin inhibitors (ATI, blue), gliadins (orange), glutenins (green) and others (grey) present in ATS from G, G-ω5, sHWP and eHWP. Percentages were determined using the intensity based absolute quantitation (iBAQ) algorithm with UPLC-TripleTOF-MS data in specific digestion mode. Digestions were performed with trypsin + chymotrypsin (TC), pepsin + trypsin + chymotrypsin (PTC), pepsin + chymotrypsin (PC) and thermolysin (TLY).
